# Optional Endoreplication and Selective Elimination of Parental Genomes during Oogenesis in Diploid and Triploid Hybrid European Water Frogs

**DOI:** 10.1371/journal.pone.0123304

**Published:** 2015-04-20

**Authors:** Dmitry Dedukh, Spartak Litvinchuk, Juriy Rosanov, Glib Mazepa, Alsu Saifitdinova, Dmitry Shabanov, Alla Krasikova

**Affiliations:** 1 Saint-Petersburg State University, Saint-Petersburg, Russia; 2 Institute of Cytology, Russian Academy of Sciences, Saint-Petersburg, Russia; 3 Department of Ecology and Genetic, Evolutionary Biology Centre, Uppsala University, Uppsala, Sweden; 4 V.N. Karazin Kharkiv National University, Kharkiv, Ukraine; Virginia Tech Virginia, UNITED STATES

## Abstract

Incompatibilities between parental genomes decrease viability of interspecific hybrids; however, deviations from canonical gametogenesis such as genome endoreplication and elimination can rescue hybrid organisms. To evaluate frequency and regularity of genome elimination and endoreplication during gametogenesis in hybrid animals with different ploidy, we examined genome composition in oocytes of di- and triploid hybrid frogs of the *Pelophylax esculentus* complex. Obtained results allowed us to suggest that during oogenesis the endoreplication involves all genomes occurring before the selective genome elimination. We accepted the hypothesis that only elimination of one copied genome occurs premeiotically in most of triploid hybrid females. At the same time, we rejected the hypothesis stating that the genome of parental species hybrid frogs co-exist with is always eliminated during oogenesis in diploid hybrids. Diploid hybrid frogs demonstrate an enlarged frequency of deviations in oogenesis comparatively to triploid hybrids. Typical for hybrid frogs deviations in gametogenesis increase variability of produced gametes and provide a mechanism for appearance of different forms of hybrids.

## Introduction

Interspecific hybridization usually leads to death or sterility of hybrid animals [1], [2]. However, in vertebrate hybrids, deviations from canonical gametogenesis and meiosis give rise to a variety of successful reproduction modes, such as parthenogenesis, gynogenesis, kleptogenesis and hybridogenesis [3–7]. Such deviations in gametogenesis include chromosomal endoreplication and elimination [8–9]. Endoreplication in germ cells leads to the formation of gametes with a multiple increase of chromosomal number [10–12]. Chromatin elimination occurring in germ cells leads to selective (in case of hybridogenesis and pre-equalizing hybrid meiosis) or nonselective (in case of kleptogenesis) deletion of genome part in gametes [5], [7], [13–15]. It remains unclear how these processes are realized during gametogenesis in hybrid animals. In particular, frequency and accuracy of genome elimination and endoreplication are poorly investigated.

Both chromosomal elimination and endoreplication take place during gametogenesis in hybrid European water frogs of the *Pelophylax esculentus* complex, which represents a widely used model for studying interspecies hybridization [14], [16–19]. This complex consists of two parental species, the lake frog *(P*. *ridibundus*, RR genotype, 2n = 26) and the pool frog *(P*. *lessonae*, LL genotype, 2n = 26), and their natural hybridogenetic form—the edible frog (*P*. *esculentus*, 2n = 26) with RL genotype [16], [17].

Not only diploid, but also two forms of triploid hybrids (with LLR and RRL genotypes, 3n = 39) exist in different *P*. *esculentus* population systems [6], [20]. *P*. *esculentus* triploids are especially abundant in the Seversky Donets river basin (Eastern Ukraine) [21–23]. Indeed, water frogs population systems found in Kharkiv region of Eastern Ukraine are represented by *P*. *ridibundus* species (R type), pure hybrid population systems (Е type), population systems of R-E type where *P*. *ridibundus* co-exists with hybrids, and rare population systems inhabited by both parental and hybrid species (R-L-Е type) [21].

In central European population systems, where *P*. *esculentus* usually co-exists with *P*. *lessonae* (L-E type), diploid hybrid frogs produce gametes with genome of *P*. *ridibundus* (R genome) [18], [19]. Previous studies of rare R-E population systems in Central Europe suggested that in diploid hybrid frogs, R genome is eliminated premeiotically while L genome is transmitted into gametes to produce hybrid frogs after crossing with parental species [6], [20]. It was thus proposed that type of transmitted genome is complementary to genome of parental species co-existing with hybrid frogs [6], [20]. We asked whether this regularity is true for *P*. *esculentus* population systems from Eastern Ukraine. We also hypothesized that in diploid hybrid frogs, endoreplication should occur during gametogenesis to produce diploid female gametes. Importantly, the diversity of chromosomal sets in gametes produced by triploid *P*. *esculentus* females has not been studied so far. Likewise, it is unknown whether chromosomes are eliminated and/or endoreplicated within the germ line in triploid frogs. Our additional aim was to check whether in triploid hybrid females single copied genome is eliminated premeiotically while double copied genome forms bivalents.

Cytogenetic analyses of germ cell karyotype in hybrid frogs reveals the changes in chromosomal number in gametogenesis, as well as the origin of diploid and triploid hybrids in different population systems. In amphibian females, parental chromosomes identification is possible by the analysis of giant lampbrush chromosomes (LBCs) obtained from growing oocytes [10], [19], [24], [25].

Lampbrush chromosomes from European water frogs were characterized in 1972 [26]. However reliable species identification was not performed and it was unclear whether analyzed frogs referred to *P*. *esculentus* complex [6], [27]. In 1979 Graf and Müller described lampbrush chromosomes from *P*. *esculentus* [28]. Precise identification of parental chromosomes in oocytes of hybrid animals was impossible until 1990 when Bucci and coauthors characterized LBCs of *P*. *ridibundus* and *P*. *lessonae* from Poland and pointed out dissimilarities between LBCs of parental species [19]. Analyzing morphological resemblance with LBCs of parental species the authors were able to identify chromosomes in oocytes of hybrid animals.

To test our hypotheses we examined lampbrush chromosome sets in oocytes from diploid and triploid hybrid *P*. *esculentus* frogs taken from the population systems of R-E type located in the East of the Ukraine. We found unusual chromosomal sets in growing oocytes connected with hybridogenetic way of reproduction. Mechanisms which lead to formation of oocytes with unusual chromosomal sets and contribution of female gametes to the maintenance of R-E hybrid *P*. *esculentus* population systems are discussed.

## Materials and Methods

### Samples studied

The European water frogs were sampled in the Kharkiv and Donetsk regions (Eastern Ukraine). *P*. *ridibundus* (N = 3) and *P*. *lessonae* (N = 2) individuals were collected from the Dnieper River basin in Krasnokutsk district proximate to hybrid formation centers. Hybrid females were taken from the Seversky Donets River basin. 13 triploid hybrid females with RRL genotype, 5 triploid hybrid females with LLR genotype and 9 diploid females with RL genotype were gathered from the population system of R-E type (S1 Table). All manipulations with animals were carried out in accordance with the national and international guidelines. The field studies did not involve endangered or protected species. Collected specimens are not listed in IUCN Redlist or by CITES. All specimens were collected in the regions of Ukraine, which are not considered as protected areas, thus no specific permissions were required for these locations. Techniques used to capture, tissue sampling and euthanasia sought to minimize animal suffering and were in accordance with recommendations of the Herpetological Animal Care and Use Committee (HACC) of the American Society of Ichthyologists and Herpetologists (available at: http://www.asih.org/publications). Each individual was anaesthetized by methoxyethane or submersion in a 1% solution of 3-aminobenzoic acid ethyl ester (MS 222). All procedures were approved by the Scientific Committee of the Biology Department of Saint-Petersburg State University.

### DNA flow cytometry

Genome composition of all frogs was established by measurement the DNA amount per nucleus using flow cytometer constructed at the Institute of Cytology, Russian Academy of Sciences, St. Petersburg. All animals were anesthetized MS222 1.5 g/l (Sigma) to take blood from the femoral vein. 0.1% Triton X100, 20 μg/ml ethidium bromide and 15 mM MgCl_2_ were added to blood samples. Blood of grass frog (*Rana temporaria* Linnaeus, 1758) and male domestic mouse (*Mus musculus*; spleenocytes, C57B1 line) were used as reference standards as published previously [29], [30]. DNA histograms were created using the formula: DNA content = (samples mean peak)/(reference standard peak) × (reference standard genome size).

### Preparation of mitotic metaphase chromosomes

Mitotic metaphase chromosomes were obtained from intestine of parental species and hybrid frogs using standard manipulations. Intestinal tissue was dissected after injection of additional animals of both parental species and hybrids with 0.2–0.5 ml of a 0.3% solution of colchicine. Intestine was incubated in 0.05 M KCl for 20 minutes, then fixed in 3:l ethanol-glacial acetic acid, where it was stored until slide preparation. Prior to metaphase plates preparation, intestine fragment was placed into a drop of 60% glacial acetic acid for 5 min and crushed. The cell suspension was dropped onto specimen slides previously heated to 60°C.

### Lampbrush chromosomes isolation

Lampbrush chromosomes were microsurgically isolated from *P*. *esculentus* oocytes according to standard procedure [31]. All females used in lampbrush chromosome analysis were not injected by colchicine or hormonal drugs. Prior to ovary isolation, frogs were anaesthetized with MS222 1.5 g/l (Sigma). Pieces of ovary were cut off from females and kept in the OR2 saline (82.5 mM NaCl, 2.5 mM KCl, 1 mM MgCl_2_, 1 mM CaCl_2_, 1 mM Na_2_HPO_4_, 5 mM HEPES (4-(2-hydroxyethyl)-1-piperazineethanesulfonic acid); pH 7.4). Nuclei were isolated from oocytes in the isolation medium “5:1” (83 mM KCl, 17 mM NaCl, 6.5 mM Na_2_HPO_4_, 3.5 mM KH_2_PO_4_, 1 mM MgCl_2_, 1 mM DTT (dithiothreitol); pH 7.0–7.2) by jeweler forceps under the observation at Leica MZ16 stereomicroscope. Each nucleus was transferred into chamber attached to a specimen slide filled with one-fourth strength “5:1” medium with the addition of 0.1% paraformaldehyde and 0.01% 1 M MgCl_2_ where nuclear envelopes were removed. Then slide preparations were centrifuged for 30 min at +4°C, 4000 rpm, fixed in 2% paraformaldehyde in 1x phosphate buffered saline (PBS) for 30 min at RT, and post-fixed in 50% ethanol for 5 min and 70% ethanol overnight (at +4°C). Preparations were not dried before immunostaining but were dehydrated in 96% ethanol for 5 min and air dried before cytological observation or FISH procedures.

### Fluorescence *in situ* hybridization

FISH with telomeric probe was carried out on lampbrush and metaphase chromosomes as described previously [32]. Metaphase plates were pre-treated with RNase A (100–200 μg/ml) for 1 h, pepsin (0.01% in 0.01 N HCl) for 10 min and then post-fixed in formaldehyde (1% in PBS, 50 mM MgCl_2_) for 10 min. Single-stranded oligonucleotide telomeric probes (TTAGGG)_5_ conjugated with Cy3 or biotin were added to hybridization mixture (40% formamide, 2.4 x SSC, and 12% dextran sulphate, 5 ng/μl labelled probe and 10–50-fold excess of tRNA). Metaphase and lampbrush chromosomes were denatured under a coverslip at 82 C for 5 min. Then slides were incubated with hybridization mixture at room temperature for 12–18 h. After hybridization, slides were washed three times in 2 x SSC at 42°C. Biotin labelled oligonucleotide probes were detected by avidin conjugated with Cy3 (Jackson ImmunoResearch Laboratories). After FISH chromosomal preparations were mounted in DABCO antifade solution containing 1 mg/ml DAPI.

### Immunofluorescent staining of lampbrush chromosomes

Immunostaining of lampbrush chromosomes spreads was performed as previously described [32], [33]. For immunostaning we used mouse monoclonal antibodies K121 against 2,2,7-trimethyl guanosine cap (dilution 1:150; Santa Cruz Biotechnology) and rabbit polyclonal antibodies H-300 against coilin (dilution 1:100; Santa Cruz Biotechnology). Lampbrush chromosome spreads were placed in 70%, 50%, 30% ethanol and in PBS with 0.01% Tween-20 for 5 min and blocked in PBS containing 1% blocking reagent (Roche) for 1 h at RT. Slides were incubated with primary antibody for 1 h at RT then washed in PBS, 0.05% Tween-20. The following secondary Abs were used: Cy3-conjugated goat anti-rabbit IgG (dilution 1:500) and Alexa-488-conjugated goat anti-mouse IgG (dilution 1:300; Jackson ImmunoResearch Laboratories). Slides were washed in PBS, 0.05% Tween-20, dehydrated in ethanol series (50%, 70%, 96%) for 5 min, air-dried and mounted in DABCO antifade solution containing 1 mg/ml DAPI.

### Wide-field microscopy

Metaphase and lampbrush chromosomes were examined using Leica fluorescence microscope DM4000 equipped with a monochrome digital camera DFC350 FX and appropriate filter cubes (Leica Wetzlar GmbH, Germany). Images were taken with 10x, 20x, 40x/1 and 100x/1.30 objectives. Leica CW 4000 FISH software was used for acquisition and processing the multicolor images.

### Confocal laser scanning microscopy

For confocal microscopy, nuclei were isolated from oocytes of 0.5–1.5 mm in diameter by jeweler forceps in the isolation medium “5:1” (described above) under the observation at Leica MZ16 stereomicroscope. Isolated nuclei were incubated for 5 min in “5:1” medium containing 0.07 μM Sytox Green (Molecular Probes) [34]. Confocal laser scanning microscopy was carried out with a Leica TCS SP5 microscope based on a Leica DMI 6000 CS inverted microscope. Specimens were examined by the XYZ scanning technique using HC PL APO 20× objective and argon laser (496 nm). Images were obtained using LAS AF software (Leica Microsystems, Germany), and 3D reconstruction was processed with Imaris 5.0.1 (Bitplane, AG) software.

## Results

### Genome composition in somatic cells of di- and triploid *P*. *esculentus* females

In this study we analyzed 27 *P*. *esculentus* females taken from population systems of R-E type in the Seversky Donets river basin (Eastern Ukraine). The differences between nuclear DNA content of *P*. *ridibundus* and *P*. *lessonae* allowed to measure the genome ploidy and to identify the precise genomic composition of hybrid frogs [21], [29] (S1 Table). The individuals with a range of C-values between 16.00±35 were designated as *P*. *ridibundus*, individuals with a range of C-values between 14.00±35 were designated as *P*. *lessonae*, while individuals with a range of C-values between 14.90±35 were designated as *P*. *esculentus* and triploid hybrid frogs with LLR and RRL genotypes have range of C-values between 21.80±35 and 22.9±35 correspondingly [21]. Among analyzed *P*. *esculentus* females there were 9 diploid (genome composition RL) and 18 triploid (genome composition RRL and LLR) animals. Two frogs had intermediate values of nuclear DNA content (designated as RLX genotype) between corresponding values of nuclear DNA content for LLR and RRL genotypes (S1 Table). Nevertheless unusual types of genomes in oocytes produced by these two frogs allowed us to refer them to RRL genotype (see below).

### Genome composition in oocytes of triploid hybrid frogs with RRL genotype

**Fig 1:**
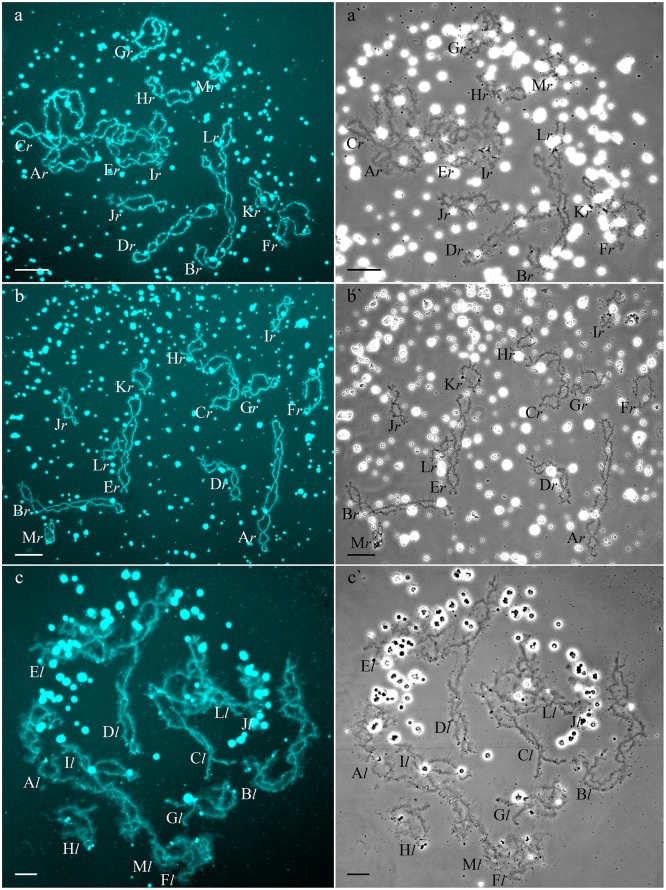
Typical lampbrush chromosome sets from oocytes of triploid hybrid frogs with RRL and LLR genotypes and diploid hybrid frog with RL genotype. Full lampbrush chromosome sets from oocytes of triploid hybrid frog with RRL (a,a`) and LLR (c,c`) genotypes and diploid hybrid frog with RL genotype (b,b`). Chromosome sets are represented by 13 bivalents, which have distribution of marker structures corresponding to *P*. *ridibundus* (a,a`,b,b`) or *P*. *lessonae* (c,c`) lampbrush chromosomes. Letter symbols indicate alphabetic numbering of all lampbrush chromosomes; italic type shows correspondence of identified chromosomes to genotype of parental species: *r*—to *P*. *ridibundus*, *l*—to *P*. *lessonae*. Chromosomes were counterstained with DAPI (a,b,c). Corresponding phase-contrast micrographs are shown (a`,b`,c`). Scale bars = 50 μm.

In R-E population system, we analyzed genome composition in oocytes of 9 diploid, 13 triploid *P*. *esculentus* females with RRL genotype (including females with RLX genotype) and 5 triploid females with LLR genotype. Algorithm to identify the type of genome transmitted in oocytes of hybrid frogs is represented in S1 Material. At first, we describe the chromosomal sets in oocytes of triploid frogs with RRL genotype. We found that 11 females with RRL genotype produced only one type of oocytes with 13 bivalents corresponding to *P*. *ridibundus* bivalents (Figs 1a,a` and 2d1–d6`; S1 Movie). Combining the data on hybrid females ploidy and oocyte genome composition, we evaluated appearance of genome elimination and endoreplication events during gametogenesis. We suggest that to form typical oocytes with 13 bivalents corresponding to *P*. *ridibundus* bivalents, elimination of L genome occurred premeiotically while two remaining R genomes presumably conjugated during meiosis (Fig 3a).

**Fig 2:**
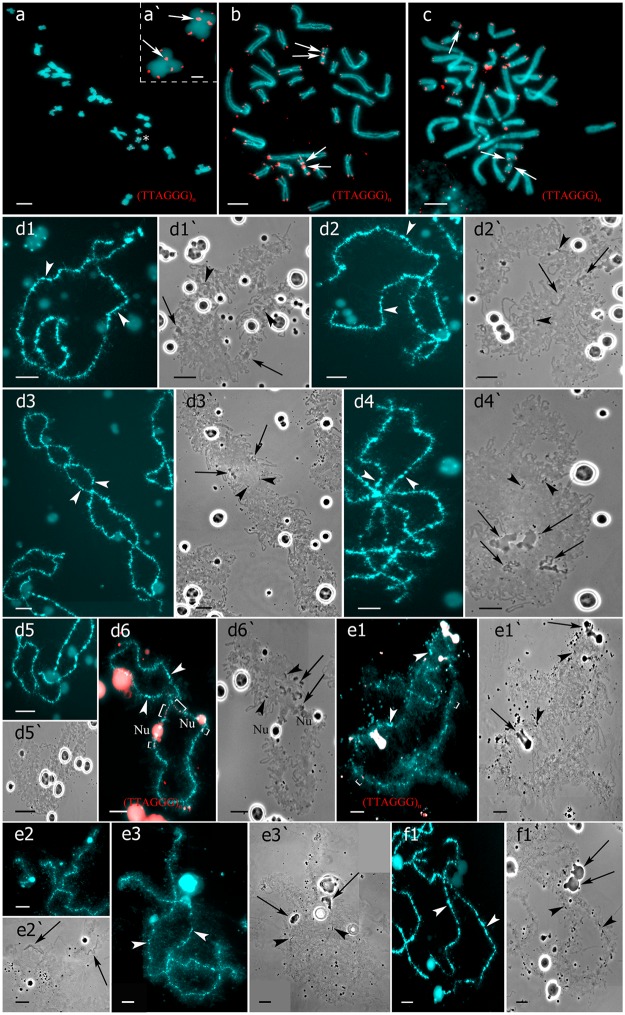
Interstitial (TTAGGG)_n_ repeat sites mapping allows to identify parental chromosomes in oocytes of hybrid frogs. (a-c) FISH mapping of (TTAGGG)_n_ repeat on metaphase chromosomes of *P*. *lessonae* (a, a`), *P*. *ridibundus* (b), and diploid *P*. *esculentus* (c). One or two interstitial (TTAGGG)_n_ repeat sites distinguish parental NOR-bearing chromosomes H (arrows). Asterisks indicate enlarged fragment with two NOR-bearing chromosomes of *P*. *lessonae*. Arrows indicate interstitial (TTAGGG)_n_ repeat sites. (d1–f1`) Lampbrush chromosomes from oocytes of triploid hybrid frogs with RRL (d1–d6`) and LLR (e1–f1`) genotypes. FISH mapping of (TTAGGG)_n_ repeat revealed lampbrush chromosome H corresponding to *P*. *ridibundus* (d6) or *P*. *lessonae* (e1) LBC H. Interstitial (TTAGGG)_n_ repeat sites are shown by square brackets. Lampbrush chromosomes corresponding to *P*. *ridibundus* LBC F (d1,d1`), G (d2,d2`), D (d3,d3`), I (d4,d4`), B (d5,d5`), and *P*. *lessonae* LBC B (e2,b2`), F (e3,b3`), L (f1,f1`) are shown. Chromosomes on micrographs (d1–d6`) were taken from the full lampbrush chromosome set represented on Fig 1a,a`. Chromosomes on micrographs (e1–e3`) were taken from the from the full lampbrush chromosome set represented on Fig 1c,c`. Various marker structures are shown by arrows. Chromosomes were counterstained with DAPI. Corresponding phase-contrast micrographs are shown (d1`,d2`,d3`,d4`,d5`,d6`,e1`,e2`,e3`,f1`). Arrowheads indicate centromeres. Scale bars = 10 μm for all panels except a`, where scale bar = 2 μm.

**Fig 3:**
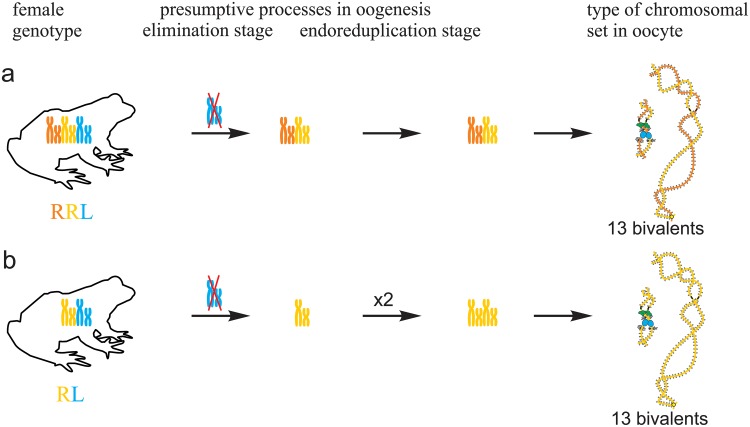
Suggested mechanisms of oogenesis typical for triploid hybrid frogs with RRL genotype and diploid hybrid frogs. (a) During oogenesis of the majority of triploid hybrids with RRL genotype from studied population systems of R-E type, L genome (blue) was eliminated while two remaining R genomes (brown and orange) without endoreplication formed 13 bivalents. (b) In oogenesis of the majority of diploid hybrids with RL genotype from studied population systems of R-E type, L genome (blue) was eliminated and the remaining R genome (orange) was endoreplicated to form 13 bivalents.

**Fig 4:**
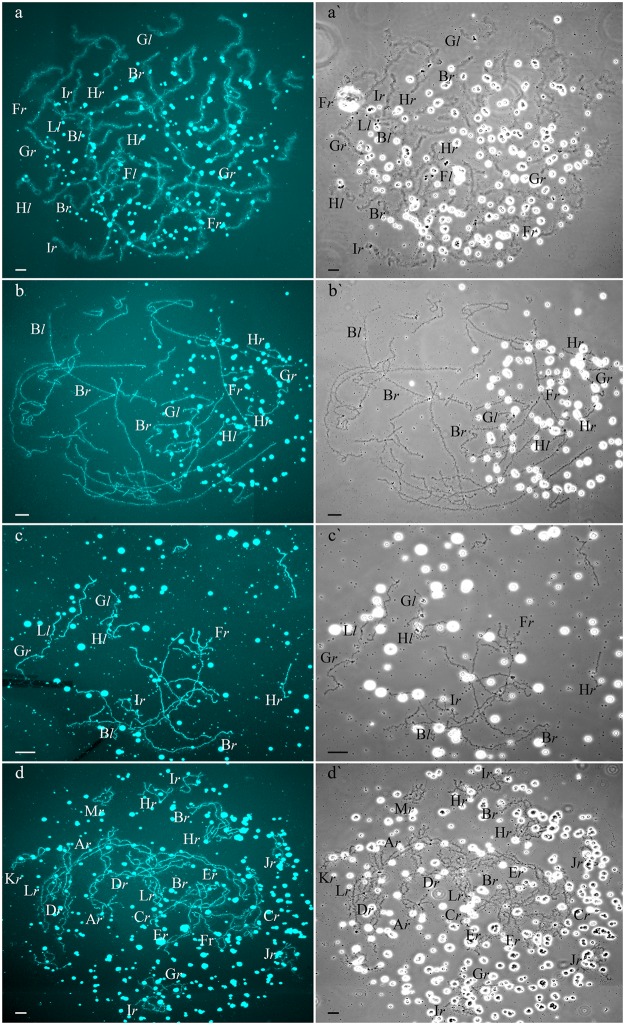
Unusual lampbrush chromosome sets from oocytes of triploid hybrid frogs with RRL genotype and two diploid hybrid frogs. (a,a`,b,b`) Lampbrush chromosome sets from oocytes of some triploid hybrid frogs with RRL genotype represented by 39 bivalents (a,a`) and 39 univalents (b,b`), with 26 bi- or univalents corresponding to *P*. *ridibundus* lampbrush chromosomes and 13 bi- or univalents corresponding to *P*. *lessonae* lampbrush chromosomes. (c,c`) Lampbrush chromosome set from oocyte of one diploid hybrid frog represented by 26 univalents. Some univalents are similar to *P*. *ridibundus* lampbrush chromosomes, while other univalents are similar to *P*. *lessonae* lampbrush chromosomes. (d,d`) Lampbrush chromosome set from oocyte of one diploid hybrid frog represented by 26 bivalents corresponding to *P*. *ridibundus* lampbrush chromosomes. Letter symbols indicate alphabetic numbering of all lampbrush chromosomes; italic type shows correspondence of identified chromosomes to genotype of parental species: *r*—to *P*. *ridibundus*, *l*—to *P*. *lessonae*. Chromosomes were counterstained with DAPI (a,b,c,d). Corresponding phase-contrast micrographs are shown (a`,b`,c`,d`). Scale bars = 50 μm.

**Fig 5:**
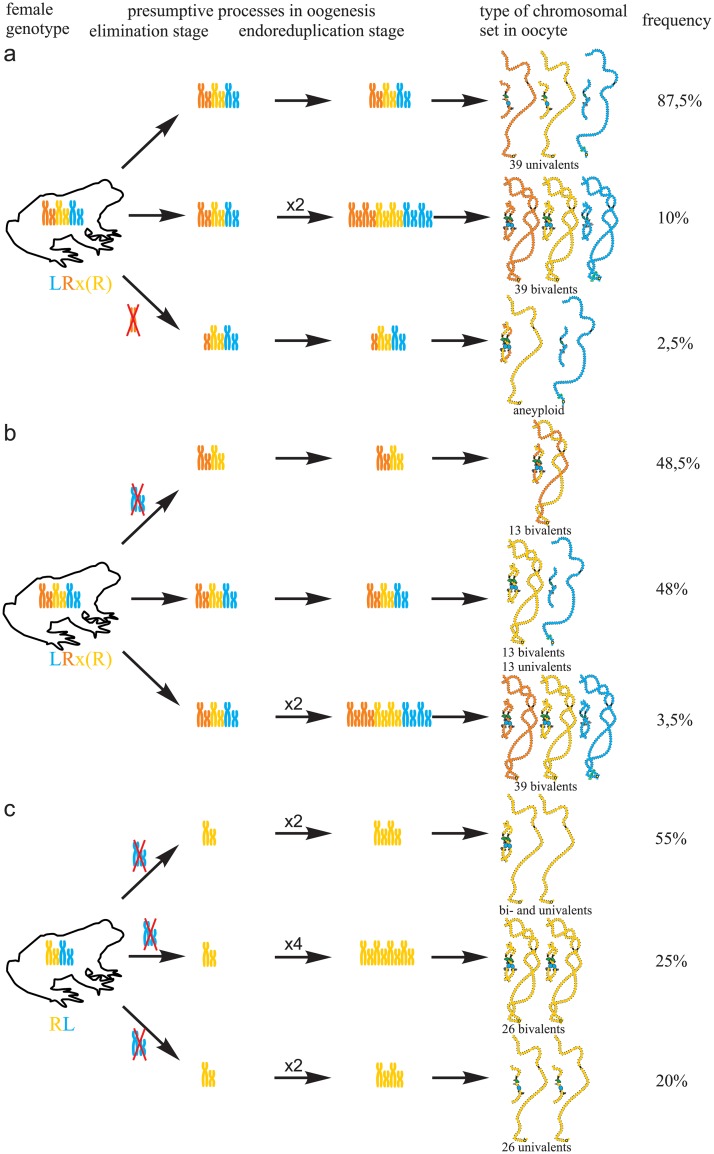
Suggested additional mechanisms of oogenesis in two triploid frogs with RRL genotype and one diploid hybrid frog. (a) During oogenesis of one triploid frog with RRL genotype neither elimination nor endoreplication occurred to form oocytes with 39 univalents (at the top), endoreplication of all genomes took place to form oocytes with 39 bivalents (in the middle), individual chromosomes from L genome (blue) were lost to form oocytes with aneuploid chromosomal sets (at the bottom). (b) During oogenesis of another triploid frog with RRL genotype elimination of L genome (blue) occurred to form oocytes with 13 bivalents (at the top), premeiotic elimination and endoreplication were absent to form oocytes with 39 univalents (in the middle), endoreplication of all genomes took place to form oocytes with 39 bivalents (at the bottom). (c) During oogenesis of one diploid hybrid frog L genome (blue) was eliminated in all observed oocytes. One round of R genome (orange) endoreplication occurred but bivalents formation was incomplete to form oocytes with both univalents and bivalents (at the top). Two rounds of endoreplication of R genome took place to form oocytes 26 bivalents (in the middle). One round of R genome endoreplication occurred but bivalents could not form that led to formation of oocytes with 26 univalents (at the bottom).

Two females with RLX genotype produced oocytes with unusual chromosomal sets. In one triploid female with RLX genotype (S1 Table), presumably RRL one, 34 oocytes contained 39 univalents, where 26 ones corresponded to *P*. *ridibundus* lampbrush chromosomes and 13 ones corresponded to *P*. *lessonae* lampbrush chromosomes (Fig 4b,b`; S1c1–d2 Fig). Importantly, 26 univalents corresponding to *P*. *ridibundus* lampbrush chromosomes did not form bivalents. Apparently, to form such oocytes neither endoreplication nor elimination occurred during gametogenesis in this triploid female (Fig 5a). In this individual, we also described 4 oocytes with 39 bivalents, where 26 ones were similar to *P*. *ridibundus* bivalents, while 13 ones were similar to *P*. *lessonae* bivalents (Fig 4a,a`; S1a1–a6`,b1–b3` Fig). Premeiotic endoreplication of the whole triploid karyotype in germ cells without any elimination is required to form oocytes with 39 bivalents (Fig 5a). One oocyte contained 8 bivalents of *P*. *ridibundus* and 15 univalents corresponding to either *P*. *ridibundus* or *P*. *lessonae* lampbrush chromosomes (S2a1–a4 and S3b,b` Figs). Such oocytes indicate abnormalities in conjugation of certain chromosomes of *P*. *ridibundus* chromosomal set. In that case, individual chromosomes of *P*. *ridibundus* were lost during oogenesis and endoreplication did not occur (Fig 5a).

For another unusual triploid with RLX genotype, most likely RRL genotype, we obtained 29 oocytes (S1 Table). We found that 13 oocytes contained 13 bivalents, identical to *P*. *ridibundus* bivalents (Fig 5b). Chromosomal sets of the other 15 oocytes from the same frog were represented by 13 bivalents corresponding to *P*. *ridibundus* lampbrush chromosomes and 13 univalents corresponding to *P*. *lessonae* lampbrush chromosomes (S3a,a` and S4b1–b4` Figs). Presumably, neither elimination nor endoreplication were required to form such oocytes during gametogenesis of triploid hybrid female with RRL genotype (Fig 5b). One oocyte from this frog contained 39 bivalents, where segregation of half-bivalents was most likely incomplete.

### Genome composition in oocytes of triploid hybrid frogs with LLR genotype

We determined the genome composition in oocytes from 5 triploid hybrid frogs with LLR genotype (S1 Table). All observed oocytes from 3 triploid hybrid frogs with LLR genotype contained 13 bivalents corresponding to *P*. *lessonae* bivalents (Figs 1c,c`, and 2e1–f1`). To produce oocytes with 13 bivalents corresponding to *P*. *lessonae* karyotype, R genome had to be eliminated premeiotically while L genome had to form bivalents (S5a Fig).

From oocytes of another triploid hybrid female with LLR genotype we obtained 18 full lampbrush chromosome sets with 13 bivalents corresponding to *P*. *ridibundus* bivalents (S6b,b` Fig). We suppose that in this hybrid frog, two L genomes were eliminated while the remaining R genome was endoreplicated premeiotically (S5b Fig).

The majority of chromosomal sets (11 of 16 sets) from the last triploid hybrid female with LLR genotype contained 26 bivalents where 13 ones were similar to *P*. *ridibundus* chromosomes and 13 ones were similar to *P*. *lessonae* chromosomes (S3d,d` Fig). Thus, during formation of these oocytes in the triploid frog, one copy of L genome must have been eliminated premeiotically, while remaining L and R genomes must have been endoreplicated premeiotically (S5c Fig). We also observed 5 lampbrush chromosome sets with 26 univalents, where 13 univalents were similar to *P*. *ridibundus* chromosomes and 13 univalents were similar to *P*. *lessonae* chromosomes (S3c,c` Fig). To form oocytes with 26 univalents only elimination occurred in germ cells of triploid frog with LLR genotype (S5c Fig).

We conclude that the majority of triploid *P*. *esculentus* females with RRL and LLR genotypes produced oocytes with 13 bivalents formed by homologous chromosomes, which are represented in double copies in genomes of triploid hybrids. Deviations in genome elimination and existence of additional endoreplication event during oogenesis in triploid hybrid frogs led to formation of oocytes with 26 and 39 bi- or univalents.

### Genome composition in oocytes of diploid hybrid frogs

All oocytes obtained from 5 typical diploid hybrid females contained 13 bivalents corresponding to *P*. *ridibundus* lampbrush chromosomes (Fig 1b,b`). We suggest that to form oocytes with 13 bivalents, L genome was eliminated while the remaining R genome was endoreplicated premeiotically in diploid hybrid frogs (Fig 3b).

Four diploid *P*. *esculentus* produced oocytes with different genome composition. In one diploid hybrid female, 20 of the 23 oocytes examined contained 26 bivalents with 13 ones corresponding to *P*. *lessonae* lampbrush karyotype and 13 ones corresponding to *P*. *ridibundus* lampbrush karyotype (S4a1–a2` and S6c,c` Figs). To form the oocytes with 26 bivalents in diploid hybrid frog during gametogenesis endoreplication of both L and R genomes was to occur (S7a Fig). Other 3 oocytes from the same frog contained 26 univalents with 13 ones corresponding to *P*. *lessonae* lampbrush chromosomes and 13 ones corresponding to *P*. *ridibundus* lampbrush chromosomes (Fig 4c,c`; S4c1–c2` Fig; S2 Movie). To form oocytes with 26 univalents in analyzed diploid hybrid frog, neither elimination nor endoreplication occurred in germ cells (S7a Fig). Previously oocytes with aneuploidy and 26 univalents corresponding to genomes of both parental species were reported for single diploid *P*. *esculentus* [19].

For another diploid frog, we obtained 6 full lampbrush chromosomal sets represented by 13 bivalents corresponding to *P*. *ridibundus* lampbrush karyotype. One oocyte from the same frog contained 26 univalents, where 13 univalents were similar to *P*. *ridibundus* lampbrush chromosomes and other 13 univalents were similar to *P*. *lessonae* lampbrush chromosomes (S7b Fig).

Among 40 oocytes with full chromosomal sets obtained from another diploid hybrid female, 28 oocytes contained 26 univalents with 13 ones corresponding to *P*. *ridibundus* chromosomes and 13 ones corresponding to *P*. *lessonae* chromosomes (S7c Fig). Other 9 oocytes contained various numbers of univalents (15 to 20) which corresponded to lampbrush chromosomes of both parental species. We suppose that aneuploid oocytes may originate after partial loss of chromosomes during gametogenesis without any endoreplication (S7c Fig). Two other sets of lampbrush chromosomes contained 26 bivalents where 13 ones were similar to *P*. *ridibundus* chromosomes, and 13 ones were similar to *P*. *lessonae* chromosomes.

In the ovary of the last atypical diploid *P*. *esculentus* we observed 8 oocytes with 26 bivalents (Fig 4d,d`; S1e1–e2` Fig), 6 oocytes with 26 univalents (S4d1–d2` and S6a,a` Figs) and 16 oocytes with various number of bivalents (from 3 to 10) and univalents (from 8 to 20). Detailed analysis revealed that all examined oocytes contained lampbrush chromosomes corresponding only to *P*. *ridibundus* karyotype. We suppose that L genome was premeiotically eliminated while R genome was premeiotically endoreplicated ones to form oocytes with 26 univalents and oocytes with both uni- and bivalents and even twice to form oocytes with 26 bivalents (Fig 5c). Alternative premeiotic endoreplication of both R and L genomes and subsequent elimination of doubled L genomes seems to be hardly possible for formation of oocytes with 13 bivalents corresponding to *P*. *ridibundus* chromosomes. In addition, we have found neither oocytes with lampbrush chromosomes corresponding to *P*. *lessonae* chromosomes nor aneuploid oocytes which are supposed to occur in such complicated way of oocytes formation. Despite oocytes with genomes of both parental species were found earlier in diploid *P*. *esculentus*, oocytes with four identical copies of one parental species genome (26 bivalents of *P*. *ridibundus*) represent unique data not only for frogs but also for other clonal animals [4], [5], [7], [15], [35], [36]. In oocytes with 26 univalents, identical chromosomes after endoreplication failed to form bivalents. Oocytes with both univalents and bivalents presumably represent unsuccessful attempts to bivalent formation (Fig 5c; S4d1–d2` and S6a,a` Figs).

Therefore, the majority of diploid *P*. *esculentus* females from Eastern Ukraine population systems of R-E type produced oocytes with 13 bivalents corresponding to *P*. *ridibundus* chromosomes. We also described diploid hybrid females with deviations in the processes of genome elimination and/or endoreplication, which led to the formation of oocytes with 26 bi- or univalents where genomes of both parental species or only genome of *P*. *ridibundus* were present.

## Discussion

### Mechanisms of oogenesis in diploid and triploid hybrid European water frogs

Transition to polyploid hybrids creates additional difficulties in gametogenesis, which require changes in the mechanisms of genome elimination and endoreplication. We are the first who represent cytogenetic observations of chromosomal sets from oocytes of triploid European water frogs. These observations are in accordance with the assumption that triploid *P*. *esculentus* females with RRL and LLR genotypes eliminate premeiotically single copied genome and produce oocytes with remaining genomes [6], [20], [35], [36] (Fig 3a; S5a Fig). 13 bivalents found in oocytes of triploid hybrids are formed between homologous chromosomes of genomes represented in two copies. Thus, our findings confirm the hypothesis suggested by Günther and co-authors in 1979 [20].

Additionally, we established that rare triploid *P*. *esculentus* females produce variable oocytes with 13 bivalents of single copied genome, oocytes with 26 uni- or bivalents, oocytes with 39 uni- or bivalents and oocytes with both bivalents and univalents (Fig 4a-b`; S3a,a`,c-d` and S6b-b` Figs). Such oocytes allowed us to evaluate occurrence of genome elimination and endoreplication during oogenesis of triploid European water frogs (Fig 5a,b; S5b,c Fig). Genome endoreplication during gametogenesis in triploid females which lead to formation of oocytes with 39 bivalents was not earlier reported for triploid frogs from other population systems. Such abnormal oocytes are common for all parthenogenetic and gynogenetic triploid vertebrate hybrids, which produce unreduced gametes and can develop without fertilization [8], [9], [37]. On the contrary, fertilization is required in the reproduction mode typical for water frogs (hybridogenesis) [16], [17], but the detailed examination of the fate of such oocytes is required. Discussion of the female gametes contribution to the maintenance of the examined European water frog population systems is represented in S2 Material.

It was expected that similar to Central European R-E population systems, in R-E population systems from Eastern Ukraine, R genome is eliminated premeiotically in diploid hybrid frogs. However, in the studied population systems of R-E type, the majority of diploid hybrid females produced oocytes with 13 bivalents of *P*. *ridibundus* (Fig 3b). We also found no support for previously suggested elimination of R genome or endoreplication of L genome during oogenesis of studied diploid hybrids. The formation of oocytes with 13 bivalents corresponding to *P*. *ridibundus* chromosomes was discovered for diploid hybrid frogs from Poland population systems of R-L-E type [19]. In contrary to triploids, in diploid hybrids bivalents consist of identical copies appeared after endoreplication event so that recombination can not increase variability in gametes of these animals.

Various deviations from obligatory elimination and endoreplication led to the formation of oocytes with 26 bi- and univalents where genomes of both parental species or only the genome of *P*. *ridibundus* appeared (Fig 5c; S7a,b,c Fig). Oocytes with 26 bivalents clearly indicate premeiotic endoreplication in diploid *P*. *esculentus* hybrids. For discussion of diploid frogs role in the maintenance of population system of R-E type see S2 Material.

We also found that the majority of di- and tripoid hybrid frogs produced oocytes with variable chromosomal sets (Fig 5a,b,c; S5c and S7a,b,c Figs). We suggest that the processes of genome elimination and endoreplication may occur independently during the development of different germ cells populations. In contrast, previously described diploid males of *P*. *esculentus* that produced both L and R haploid gametes were considered mosaic [38].

Directed genome duplication in gametogenesis widely spreads among the majority of clonal animals and normally does not happen in sexually reproduced species. In the analysed samples, genome endoreplication occurred premeiotically in few triploid and all diploid hybrid frogs and in one diploid hybrid frog even twice (Figs 3b and 5a,b,c; S5b,c and S7a,b,c Figs). In comparison to elimination event, genome duplication is unselective for any parental species genomes. According to the schemes of the suggested mechanisms of oogenesis (Figs 3b and 5c; S5b,c and S7b Figs) genome endoreplication always occurred after elimination if both processes happened during oogenesis. The omission of cell division after DNA synthesis stage of cell cycle called endoreplicaton is considered a possible mechanism of genome duplication [9], [39].

### Mechanisms of genome elimination during oogenesis in hybrid European water frogs with different ploidy

Selective genome elimination is a key mechanism leading to appearance of oocytes with one of the parental genomes in water frogs. Possible mechanisms of genome elimination in germ cells of hybrid organisms were previously suggested. It was found that chromosomes of one species may lag and vanish during single [13] or contiguous mitotic [18], [40] or single meiotic division [41]. Differences between sequences of parental species centromeres or centromere binding proteins may be responsible for chromosome loss during division [42]. However, our previous data indicate no any difference in centromere repeats in parental water frog species of geographic origin close to the studied populations [32]. Nevertheless, elimination may also occur during interphase via chromatin budding from the nucleus of germ cells and its further degradation [40], [43].

Not only centromeric but also other repetitive sequences such as transposons differ between closely related parental species [42], [44]. Divergence in transposons in parental genomes leads to the dissimilarities in noncoding RNA in germ cells of hybrid animals playing a role in heterochromatin formation [42]. The mechanism of hybrid disgenesis in *D*. *melanogaster* mediated by piRNAs selectively blocking activity of transposons from one of the parents [45] may be the mechanism of selective genome elimination in water frog hybrids. We suppose that the genome which was absent and had not transcribed noncoding RNA in maternal oocyte should be eliminated during the gametogenesis in hybrid frog arising after fertilization of this oocyte.

Selective elimination in hybrid frogs may also be similar to paternal genome elimination naturally occurring in some insects and hybridogenetic all-female fishes [46]. In these cases selective genome elimination is supposed to be based on competition between genomes of both parental species for preferential transmitting into gametes. Such genome competition could also lead to elimination-cause mutation in one genome leading to selective elimination of the other parental species genome [46]. Competition between *P*. *ridibundus* and *P*. *lessonae* genomes may also result in appearance of elimination-cause mutation more frequently in *P*. *ridibundus* than in *P*. *lessonae* genomes.

Oocyte formation in triploid *P*. *esculentus* frogs is more stable and typically includes only premeiotic genome elimination without endoreplication. Instability in chromatin elimination and appearance of endoreplication lead to formation of oocytes with genome composition being identical to twofold genome composition in somatic cells. Diploid hybrid *P*. *esculentus* females have more frequent deviations in genome elimination and endoreplication events and can produce oocytes with different genome composition and different ploidy. The majority of oocytes can participate in gamete formation and provide gamete variations required for successful reproduction of di- and triploid hybrid water frogs in different populations.

## Supporting Information

S1 Fig Individual chromosome identification in unusual lampbrush chromosome sets. Identification of individual lampbrush chromosomes from chromosome sets with 39 bivalents (a1–b3`) and 39 univalents (c1–d2`) from triploid frog with RRL genotype and sets with 26 bivalents (e1–e2`) from diploid hybrid frog. (a1–a3) Lampbrush chromosomes corresponding to *P*. *ridibundus* (a1–a2) or to *P*. *lessonae* (a3) lampbrush chromosome G. (a4–a6,b1–b3,c1,d1–d2,e1–e2) Lampbrush chromosomes corresponding to *P*. *ridibundus* (a4–a5`,b1–b2`,c1–d1`,e1–e2`) or to *P*. *lessonae* (a6,a6`,b3,b3`,d2,d2`) lampbrush chromosome H. FISH mapping of (TTAGGG)_n_ repeat. Interstitial (TTAGGG)_n_ repeat sites are shown by square brackets. Chromosomes on micrographs (a1–a6`) were taken from the full chromosome set represented on the Fig 4a,a`. Chromosomes on micrograph (b1–b3`) were taken from the other chromosome set with 39 bivalents. Lampbrush chromosomes on micrographs (c1,c1`) and (d1–d2`) were taken from different chromosome sets containing 39 univalents (full chromosome set not shown and represented on Fig 4b,b` correspondingly). Chromosomes on micrographs (e1–e2`) were taken from the full chromosome set represented on Fig 4d,d`. Various marker structures are shown by arrows. Arrowheads indicate centromeres. Chromosomes were counterstained with DAPI. Corresponding phase-contrast micrographs are shown (a`,b`,c`). Scale bars = 10 μm.
(PDF)

S2 Fig Individual chromosome identification in aneuploid lampbrush chromosome set obtained from oocytes of triploid hybrid frog with RRL genotype. (a1,a2) Bivalent corresponding to *P*. *ridibundus* lampbrush chromosome H (a1) and univalent corresponding to *P*. *lessonae* lampbrush chromosome H (a2). (a3,a4) Univalents corresponding to *P*. *ridibundus* (a3) and *P*. *lessonae* (a4) lampbrush chromosome B. All chromosomes were taken from the full lampbrush chromosome set represented in S3b,b` Fig. FISH mapping of (TTAGGG)_n_ repeat (a1–a4). Interstitial (TTAGGG)_n_ repeat sites are shown by square brackets. Chromosomes were counterstained with DAPI. Arrowheads show centromeres. Scale bars = 10 μm.
(PDF)

S3 Fig Sample lampbrush chromosome sets from oocytes produced by triploid hybrid females with RRL and LLR genotypes. (a,a`) Lampbrush chromosome set from oocyte of one triploid hybrid female with RRL genotype is represented by 13 bivalents corresponding to *P*. *ridibundus* chromosomes and 13 univalents corresponding to *P*. *lessonae* chromosomes. (b,b`) Aneuploid lampbrush chromosome set from oocyte of another triploid hybrid female with RRL genotype is represented by 9 bivalents similar to *P*. *ridibundus* lampbrush chromosomes and about 18 univalents, some of them being similar to *P*. *ridibundus* lampbrush chromosomes. (c,c`) Lampbrush chromosome set from oocyte of one triploid hybrid female with LLR genotype represented by 26 univalents, where 13 univalents correspond to *P*. *ridibundus* chromosomes and other 13 univalents correspond to *P*. *lessonae* chromosomes. (d,d`) Lampbrush chromosome set from oocyte of another triploid hybrid female with LLR genotype is represented by 26 bivalents. 13 bivalents are similar to *P*. *ridibundus* lampbrush chromosomes and 13 bivalents are similar to *P*. *lessonae* lampbrush chromosomes. Letter symbols indicate alphabetic numbering of all lampbrush chromosomes; italic type shows correspondence of identified chromosomes to genotype of parental species: *r*—to *P*. *ridibundus*, *l*—to *P*. *lessonae*. Chromosomes were counterstained with DAPI. Corresponding phase-contrast micrographs are shown (a`,b`,c`,d`). Scale bars = 50 μm.
(PDF)

S4 Fig Individual chromosome identification in lampbrush chromosome sets with univalents and bivalents. Lampbrush chromosomes from chromosome sets with 13 bivalents and 13 univalents (b1–b4`) from oocytes of triploid hybrid with RRL genotype, 26 bivalents (a1–a2`), and 26 univalents (c1–c2`and d1–d2`) from oocytes of different diploid hybrid. Lampbrush chromosomes corresponding to *P*. *ridibundus* (a1,a1`) and to *P*. *lessonae* (a2,a2`) lampbrush chromosome H were taken from full chromosome set represented on S7c,c` Fig. Bivalents G (b1,b1`) and Н (b3,b3`) are similar to *P*. *ridibundus* lampbrush chromosomes, and univalents G (b2,b2`) and H (b4,b4`) are similar to *P*. *lessonae* lampbrush chromosomes. These lampbrush chromosomes were taken from full lampbrush chromosome set represented on S3a,a` Fig. Lampbrush chromosomes corresponding to *P*. *ridibundus* (c1,c1`) and to *P*. *lessonae* (c2,c2`) lampbrush chromosome H were taken from full lampbrush chromosome set represented on Fig 4c,c`. Univalents corresponding to *P*. *ridibundus* lampbrush chromosome H (d1, d1`, d2, d2`) were taken from chromosome set represented on S6a,a` Fig. FISH mapping of (TTAGGG)_n_ repeat (a1,a2,b3,b4,c1,c2,d1,d2). Interstitial (TTAGGG)_n_ repeat sites are shown by square brackets. Chromosomes were counterstained with DAPI. Corresponding phase-contrast micrographs are shown (a1`,a2`,b3`,b4`,c1`,c2`,d1`,d2`). Arrows indicate the marker loops. Arrowheads show centromeres. Scale bars = 10 μm.
(PDF)

S5 Fig Suggested mechanisms of oogenesis in triploid hybrid frogs with LLR genotype. (a) During oogenesis of triploid hybrid frog with LLR genotype R genome (orange) was eliminated and remaining L genomes (light blue and blue) without endoreplication formed 13 bivalents. (b) During oogenesis of other triploid frog with LLR genotype both L genomes (blue, light blue) were eliminated and R genome was endoreplicated to form oocytes with 13 bivalents. (c) During oogenesis of triploid hybrid frog with LLR genotype elimination of one L genome (light blue) and endoreplication of remaining genomes occurred to form oocytes with 26 bivalents (at the top). Elimination of one L genome (light blue) without endoreplication of remaining genomes took place to form oocytes with 26 univalents (at the bottom).
(PDF)

S6 Fig Additional lampbrush chromosome sets obtained from oocytes of diploid hybrid frogs and triploid hybrid frogs with LLR genotype. (a,a`) Lampbrush chromosome set from oocyte of diploid hybrid female represented by 26 univalents corresponding to *P*. *ridibundus* chromosomes. (b,b`) Lampbrush chromosome set from oocyte of diploid hybrid female represented by 13 bivalents corresponding to *P*. *ridibundus* chromosomes. (c,c`) Lampbrush chromosome set from oocyte of triploid hybrid female with LLR genotype represented by 26 bivalents, where 13 bivalents correspond to *P*. *ridibundus* chromosomes and 13 bivalents correspond to *P*. *lessonae* chromosomes. Letter symbols indicate alphabetic numbering of all lampbrush chromosomes; italic type shows correspondence of identified chromosomes to genotype of parental species: *r*—to *P*. *ridibundus*, *l*—to *P*. *lessonae*. Chromosomes were counterstained with DAPI. Corresponding phase-contrast micrographs are shown (a`,b`,c`). Scale bars = 50 μm.
(PDF)

S7 Fig Suggested additional mechanisms of oogenesis in diploid hybrid frogs. (a) During oogenesis of diploid hybrid frog only endoreplication of both parental genomes occurred to form oocytes with 26 bivalents (at the top), neither elimination nor endoreplication took place to form oocytes with 26 univalents (at the bottom). (b) During oogenesis of other diploid hybrid frog elimination of L genome (blue) and endoreplication of the remaining R genome (orange) occurred to form oocytes with 13 bivalents (at the top), elimination and endoreplication were omitted to form oocytes with 26 univalents (at the bottom). (c) During oogenesis of additional diploid hybrid frog neither elimination nor endoreplication occurred to form oocytes with 26 univalents (at the top). Losing of individual chromosomes corresponding to *P*. *ridibundus* chromosomes led to formation of aneuploid oocytes (in the middle). Endoreplication took place to form oocytes with 26 bivalents (at the bottom).
(PDF)

S8 Fig Ovaries of parental species and hybrid *P*. *esculentus* frogs. Ovary fragments of *P*. *ridibundus* (a), *P*. *lessonae* (b), triploid hybrid frog with LLR genotype (c) and diploid hybrid frog (d). Pre-vitellogenic, vitellogenic and post-vitellogenic oocytes (according to Dumont (1972) [*]) are present in the mature ovaries (a,c) but only pre- and vitellogenic oocytes are present in the immature ovaries (b,d). Ovaries of both parental species (a,b) are characterized by alive oocytes with regular rounded shape and a few dead oocytes. Ovaries of hybrid animals (c,d) have many dead oocytes with irregular shape and abnormal dark brown coloring of oocyte poles. Scale bars = 1 mm. * Dumont JN (1972) Oogenesis in *Xenopus laevis* (Daudin). I. Stages of oocyte development in laboratory maintained animals. J Morphol 136: 153–180.
(PDF)

S9 Fig Marker structures on lampbrush chromosomes from hybrid frogs. (a) Detection of chromosome-associated coilin-positive granules by immunofluorescent staining with R288 antibody. (b) Identification of marker loops enriched with splicing factors on lampbrush chromosome corresponding to chromosome I of *P*. *ridibundus*. Immunofluorescent staining with antibodies against TMG-cap of small nuclear RNA. Arrows show marker loops. Arrowheads indicate centromeres. Chromosomes were counterstained with DAPI. Corresponding phase-contrast micrographs are shown (a`,b`). Scale bars = 10 μm.
(PDF)

S10 Fig Chromosome sets of oocytes from females with different genotypes presumably contributing in maintenance of R-E type population systems. Triploid hybrids with RRL genotype produce oocytes with 13 bivalents corresponding to *P*. *ridibundus* chromosomes (at the top). Triploid females with LLR genotype produce oocytes with 13 bivalents corresponding to *P*. *lessonae* chromosomes, oocytes with 13 bivalents corresponding to *P*. *ridibundus* chromosomes and oocytes with 26 bivalents corresponding to both *P*. *ridibundus* and *P*. *lessonae* chromosomes (in the middle). Diploid hybrid frogs produce oocytes with 13 bivalents corresponding to *P*. *ridibundus* chromosomes, oocytes with 26 bivalents corresponding to both *P*. *ridibundus* and *P*. *lessonae* chromosomes and oocytes with 26 bivalents corresponding only to *P*. *ridibundus* chromosomes (at the bottom).
(PDF)

S1 Material Description of ovaries from parental species and hybrid frogs and algorithm of oocytes karyotype identification. (DOC)

S2 Material Mechanisms of hybrid frogs reproduction in the studied population systems of R-E type. (DOC)

S1 Movie 3D projection of the intact oocyte nucleus from diploid hybrid *P*. *esculentus* containing lampbrush chromosome set represented by 13 bivalents. (AVI)

S2 Movie 3D projection of the intact oocyte nucleus from diploid hybrid *P*. *esculentus* containing lampbrush chromosome set represented by 26 univalents. (AVI)

S1 Table List of *P*. *esculentus* females from population systems of R-E type from the Seversky Donets river basin in Eastern Ukraine. C values—the amount of DNA per nucleus (genome size, in picograms, pg)—and genotypes are given for each female.
(PDF)
