# Supplementary figures and images for: Optional Endoreplication and Selective Elimination of Parental Genomes during Oogenesis in Diploid and Triploid Hybrid European Water Frogs
Source: PLoS One. 2015 Apr 20;10(4):e0123304. doi: 10.1371/journal.pone.0123304 (PMC4403867; doi:10.1371/journal.pone.0123304)

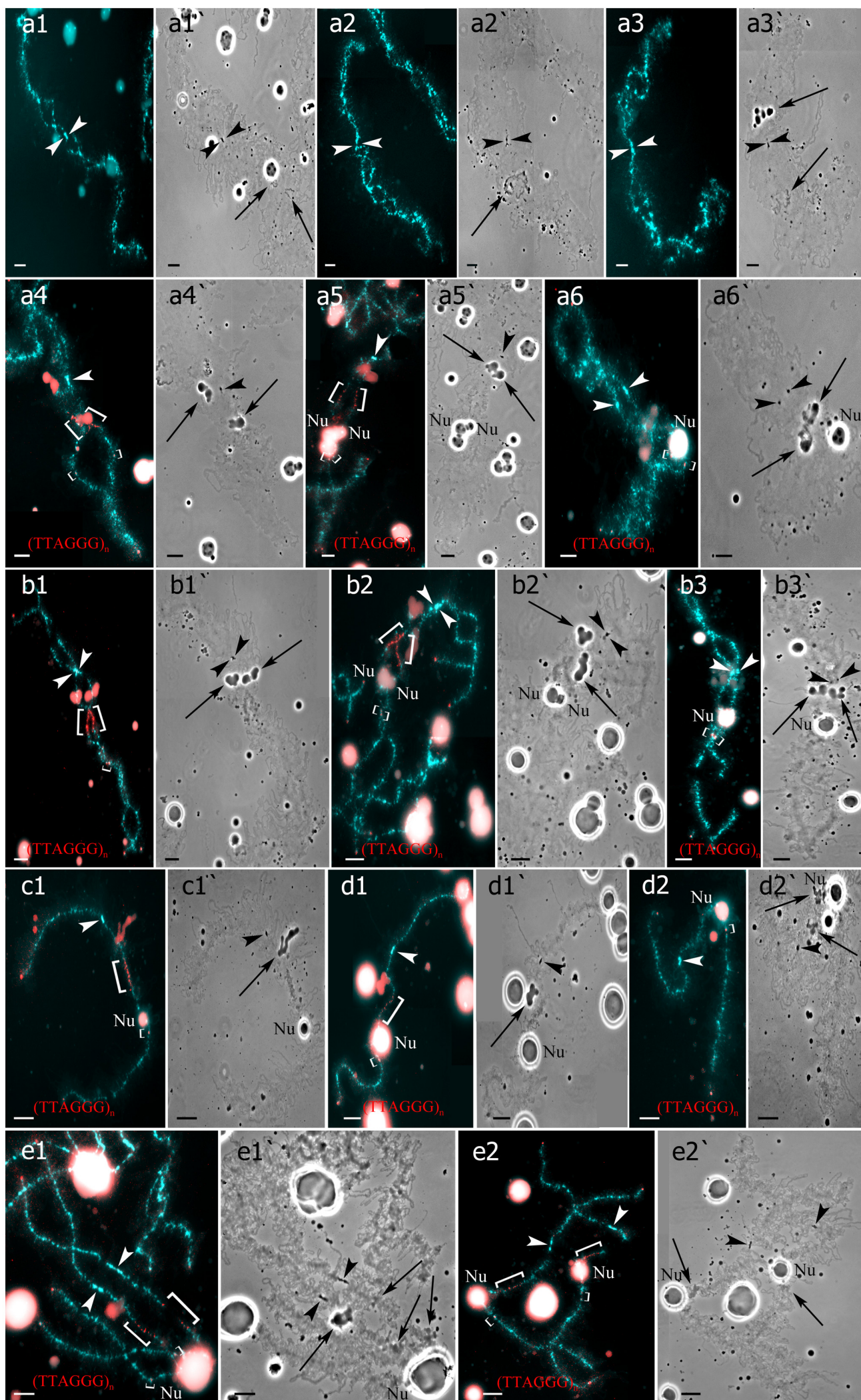

Supplement: S1 Fig — Identification of individual lampbrush chromosomes from chromosome sets with 39 bivalents (a1–b3`) and 39 univalents (c1–d2`) from triploid frog with RRL genotype and sets with 26 bivalents (e1–e2`) from diploid hybrid frog. (a1–a3) Lampbrush chromosomes corresponding to P. ridibundus (a1–a2) or to P. lessonae (a3) lampbrush chromosome G. (a4–a6,b1–b3,c1,d1–d2,e1–e2) Lampbrush chromosomes corresponding to P. ridibundus (a4–a5`,b1–b2`,c1–d1`,e1–e2`) or to P. lessonae (a6,a6`,b3,b3`,d2,d2`) lampbrush chromosome H. FISH mapping of (TTAGGG)n repeat. Interstitial (TTAGGG)n repeat sites are shown by square brackets. Chromosomes on micrographs (a1–a6`) were taken from the full chromosome set represented on the Fig 4a,a`. Chromosomes on micrograph (b1–b3`) were taken from the other chromosome set with 39 bivalents. Lampbrush chromosomes on micrographs (c1,c1`) and (d1–d2`) were taken from different chromosome sets containing 39 univalents (full chromosome set not shown and represented on Fig 4b,b` correspondingly). Chromosomes on micrographs (e1–e2`) were taken from the full chromosome set represented on Fig 4d,d`. Various marker structures are shown by arrows. Arrowheads indicate centromeres. Chromosomes were counterstained with DAPI. Corresponding phase-contrast micrographs are shown (a`,b`,c`). Scale bars = 10 μm. (PDF) [file pone.0123304.s001.pdf]

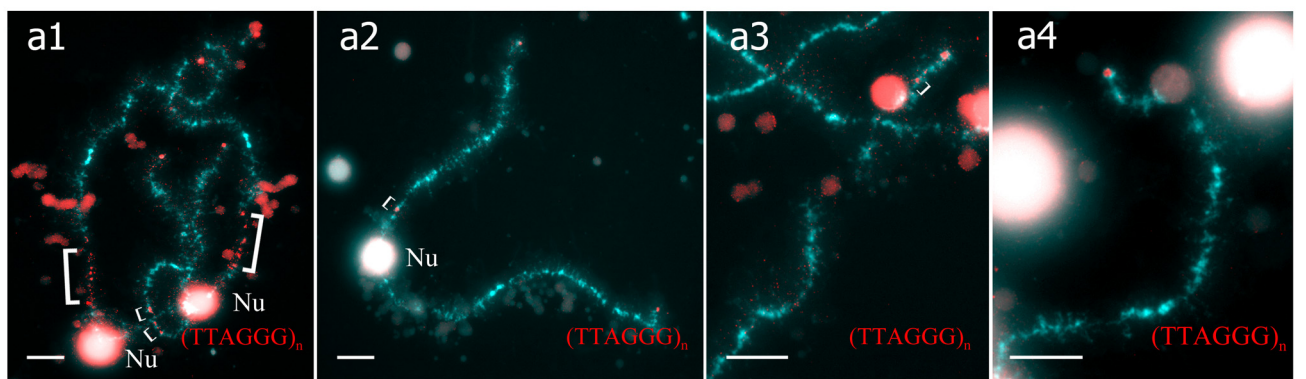

Supplement: S2 Fig — (a1,a2) Bivalent corresponding to P. ridibundus lampbrush chromosome H (a1) and univalent corresponding to P. lessonae lampbrush chromosome H (a2). (a3,a4) Univalents corresponding to P. ridibundus (a3) and P. lessonae (a4) lampbrush chromosome B. All chromosomes were taken from the full lampbrush chromosome set represented in S3b,b` Fig. FISH mapping of (TTAGGG)n repeat (a1–a4). Interstitial (TTAGGG)n repeat sites are shown by square brackets. Chromosomes were counterstained with DAPI. Arrowheads show centromeres. Scale bars = 10 μm. (PDF) [file pone.0123304.s002.pdf]

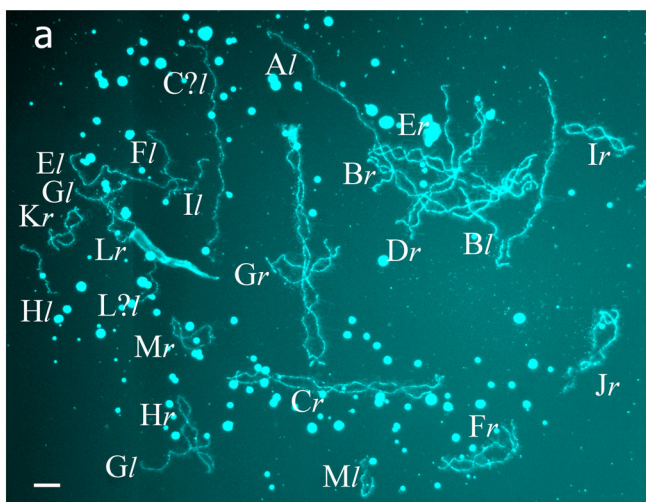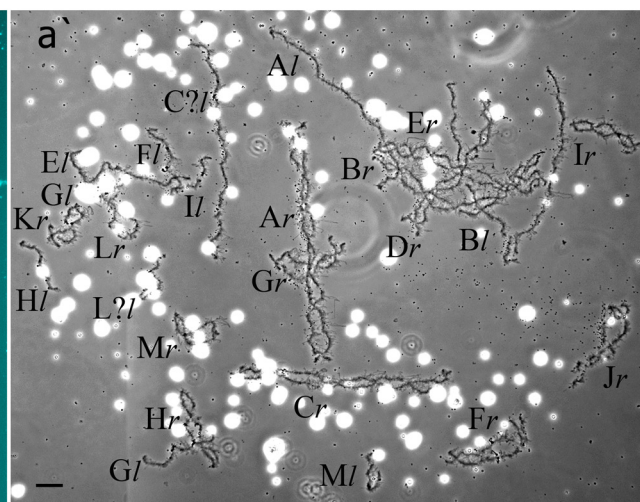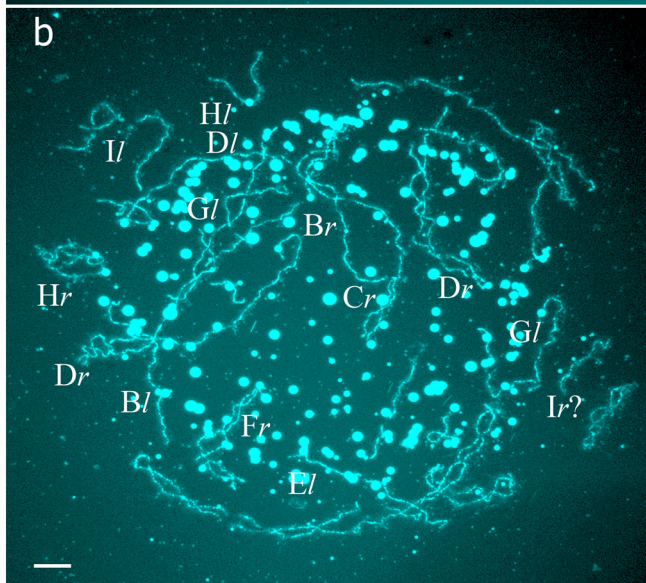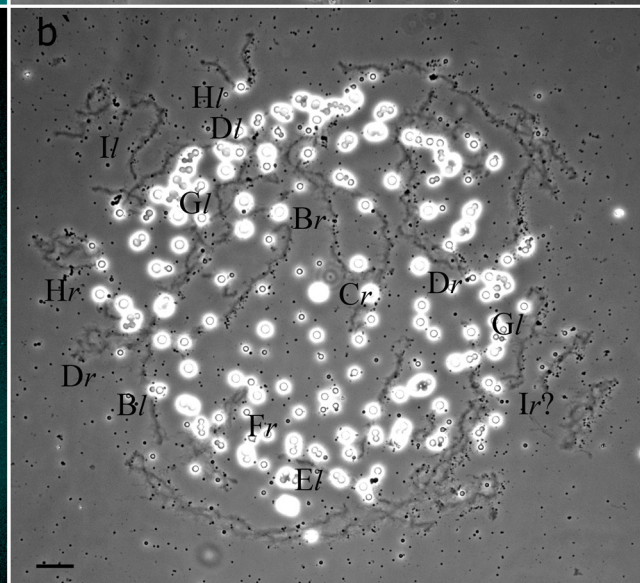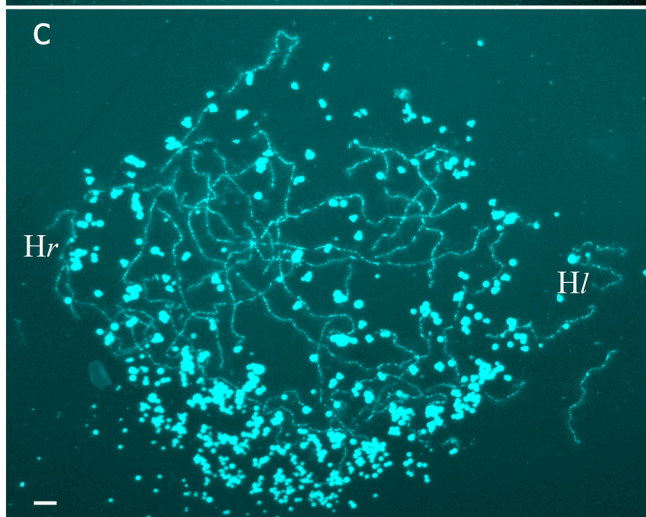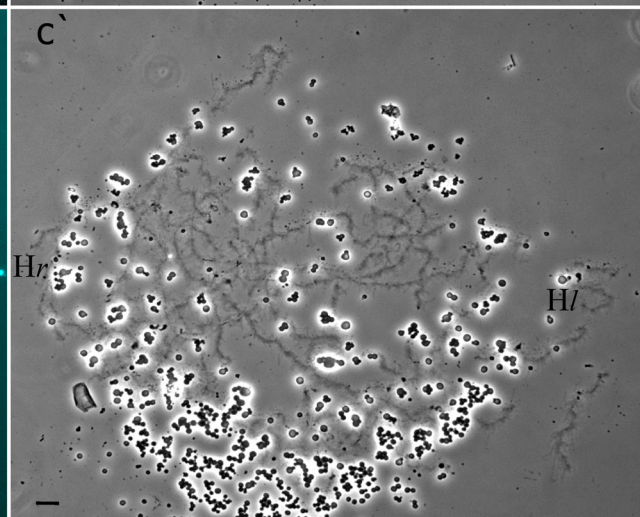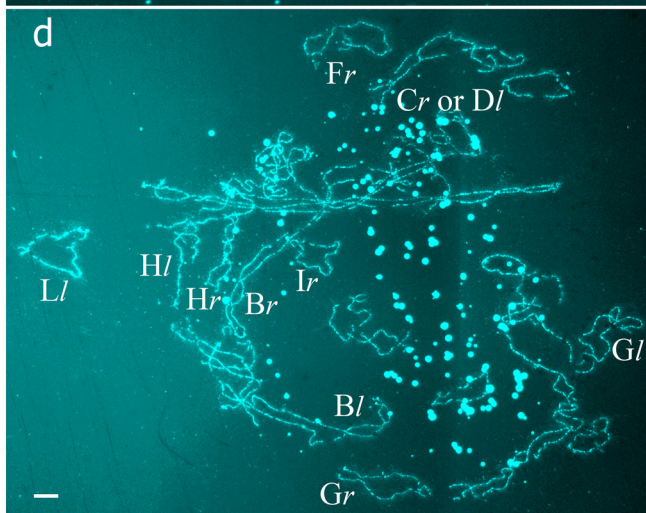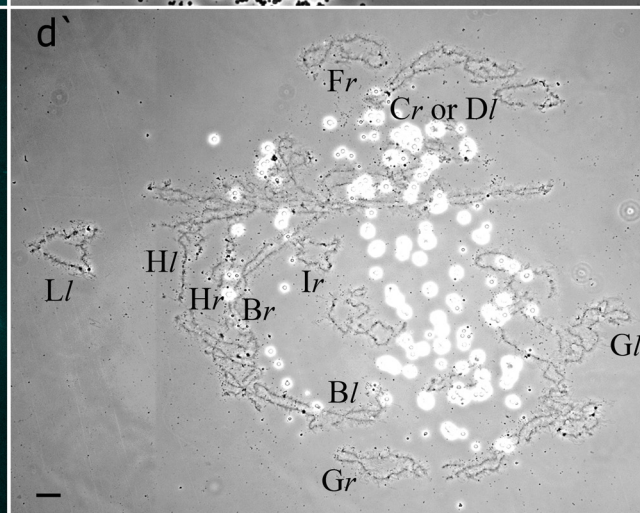

Supplement: S3 Fig — (a,a`) Lampbrush chromosome set from oocyte of one triploid hybrid female with RRL genotype is represented by 13 bivalents corresponding to P. ridibundus chromosomes and 13 univalents corresponding to P. lessonae chromosomes. (b,b`) Aneuploid lampbrush chromosome set from oocyte of another triploid hybrid female with RRL genotype is represented by 9 bivalents similar to P. ridibundus lampbrush chromosomes and about 18 univalents, some of them being similar to P. ridibundus lampbrush chromosomes. (c,c`) Lampbrush chromosome set from oocyte of one triploid hybrid female with LLR genotype represented by 26 univalents, where 13 univalents correspond to P. ridibundus chromosomes and other 13 univalents correspond to P. lessonae chromosomes. (d,d`) Lampbrush chromosome set from oocyte of another triploid hybrid female with LLR genotype is represented by 26 bivalents. 13 bivalents are similar to P. ridibundus lampbrush chromosomes and 13 bivalents are similar to P. lessonae lampbrush chromosomes. Letter symbols indicate alphabetic numbering of all lampbrush chromosomes; italic type shows correspondence of identified chromosomes to genotype of parental species: r—to P. ridibundus, l—to P. lessonae. Chromosomes were counterstained with DAPI. Corresponding phase-contrast micrographs are shown (a`,b`,c`,d`). Scale bars = 50 μm. (PDF) [file pone.0123304.s003.pdf]

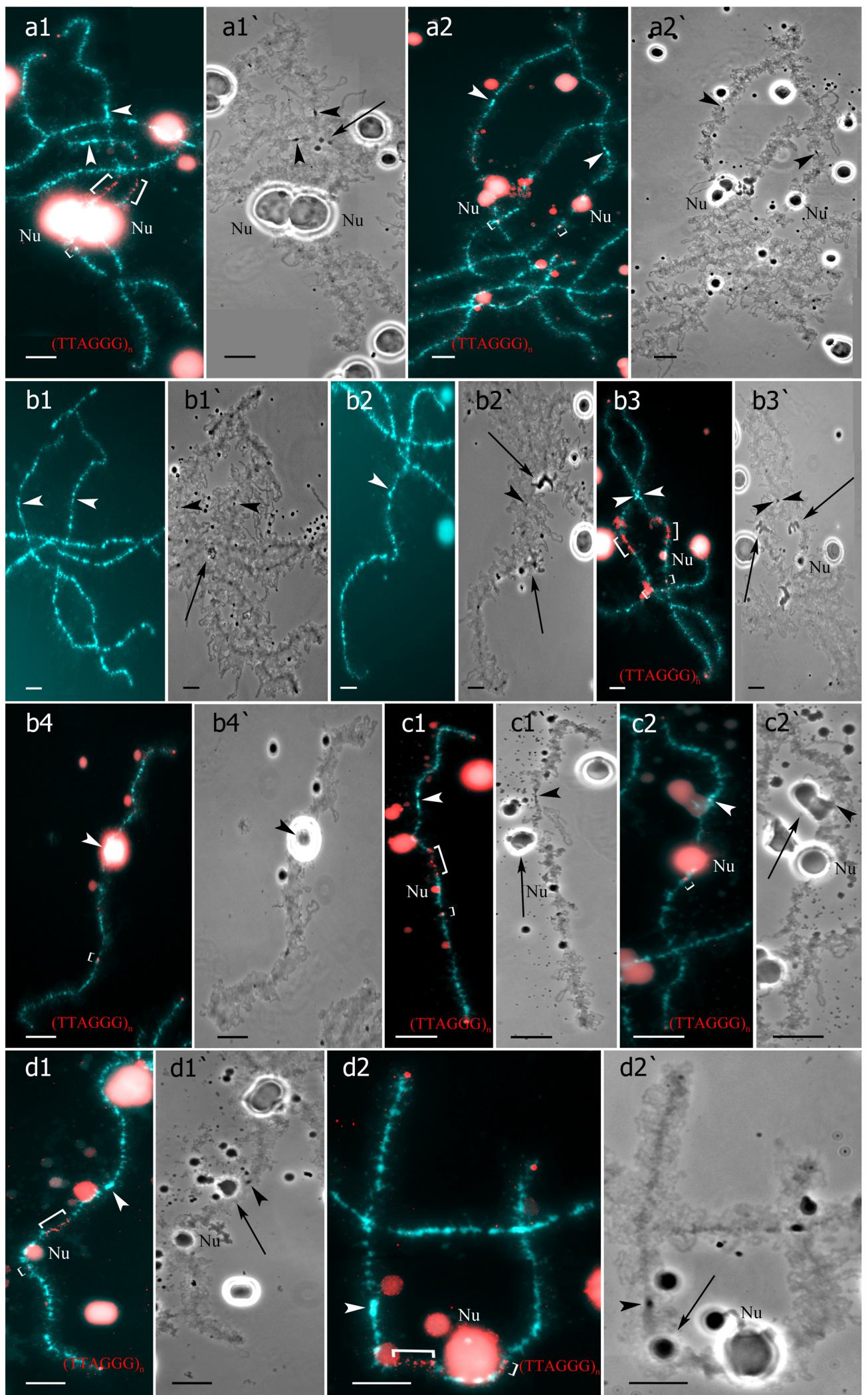

Supplement: S4 Fig — Lampbrush chromosomes from chromosome sets with 13 bivalents and 13 univalents (b1–b4`) from oocytes of triploid hybrid with RRL genotype, 26 bivalents (a1–a2`), and 26 univalents (c1–c2`and d1–d2`) from oocytes of different diploid hybrid. Lampbrush chromosomes corresponding to P. ridibundus (a1,a1`) and to P. lessonae (a2,a2`) lampbrush chromosome H were taken from full chromosome set represented on S7c,c` Fig. Bivalents G (b1,b1`) and Н (b3,b3`) are similar to P. ridibundus lampbrush chromosomes, and univalents G (b2,b2`) and H (b4,b4`) are similar to P. lessonae lampbrush chromosomes. These lampbrush chromosomes were taken from full lampbrush chromosome set represented on S3a,a` Fig. Lampbrush chromosomes corresponding to P. ridibundus (c1,c1`) and to P. lessonae (c2,c2`) lampbrush chromosome H were taken from full lampbrush chromosome set represented on Fig 4c,c`. Univalents corresponding to P. ridibundus lampbrush chromosome H (d1, d1`, d2, d2`) were taken from chromosome set represented on S6a,a` Fig. FISH mapping of (TTAGGG)n repeat (a1,a2,b3,b4,c1,c2,d1,d2). Interstitial (TTAGGG)n repeat sites are shown by square brackets. Chromosomes were counterstained with DAPI. Corresponding phase-contrast micrographs are shown (a1`,a2`,b3`,b4`,c1`,c2`,d1`,d2`). Arrows indicate the marker loops. Arrowheads show centromeres. Scale bars = 10 μm. (PDF) [file pone.0123304.s004.pdf]

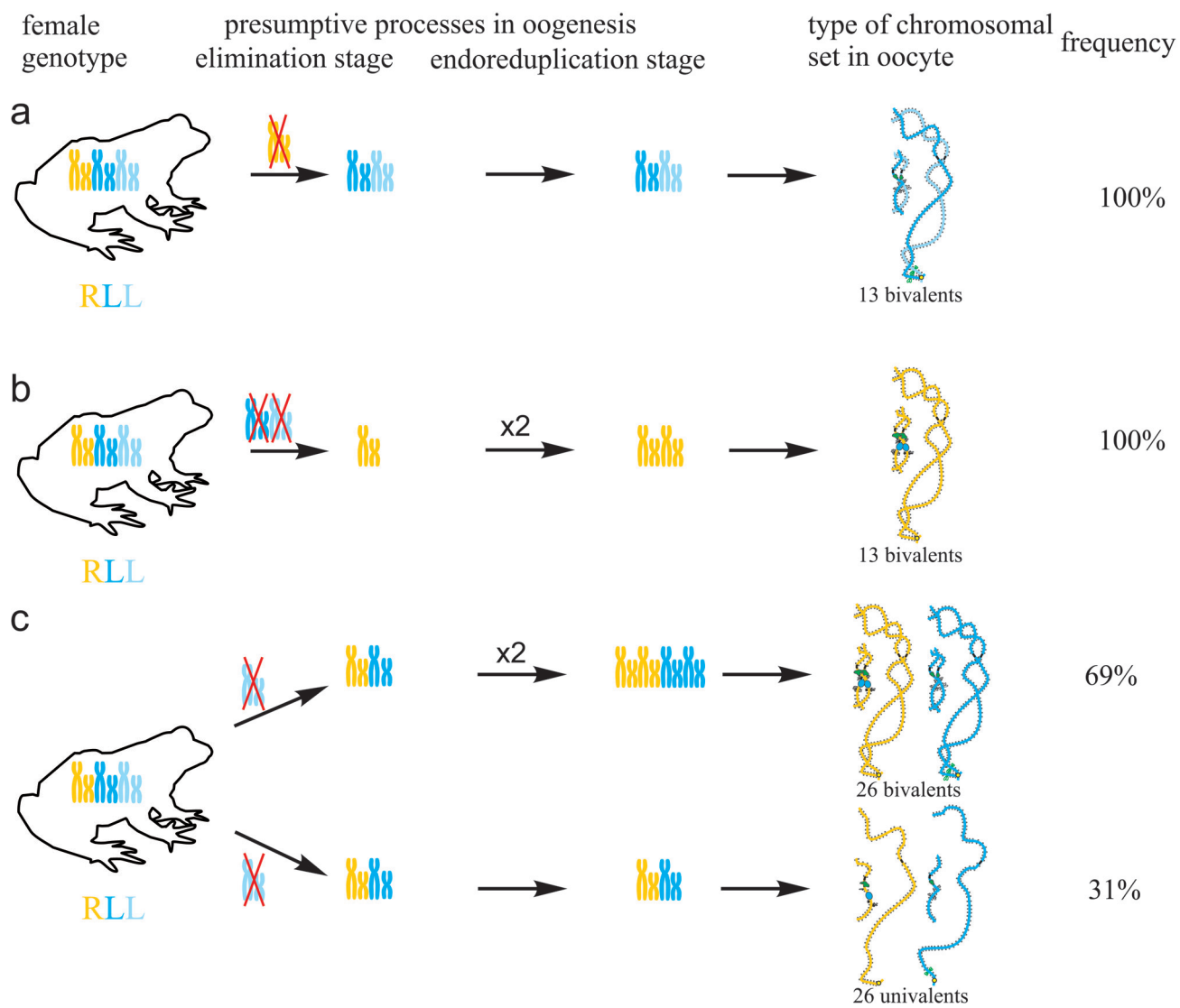

Supplement: S5 Fig — (a) During oogenesis of triploid hybrid frog with LLR genotype R genome (orange) was eliminated and remaining L genomes (light blue and blue) without endoreplication formed 13 bivalents. (b) During oogenesis of other triploid frog with LLR genotype both L genomes (blue, light blue) were eliminated and R genome was endoreplicated to form oocytes with 13 bivalents. (c) During oogenesis of triploid hybrid frog with LLR genotype elimination of one L genome (light blue) and endoreplication of remaining genomes occurred to form oocytes with 26 bivalents (at the top). Elimination of one L genome (light blue) without endoreplication of remaining genomes took place to form oocytes with 26 univalents (at the bottom). (PDF) [file pone.0123304.s005.pdf]

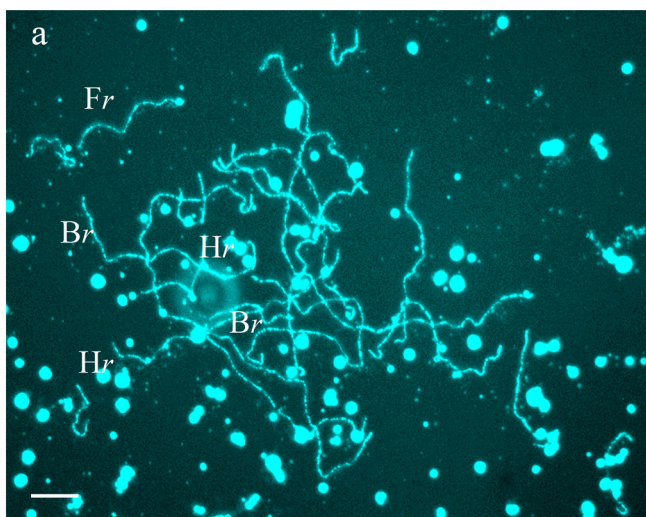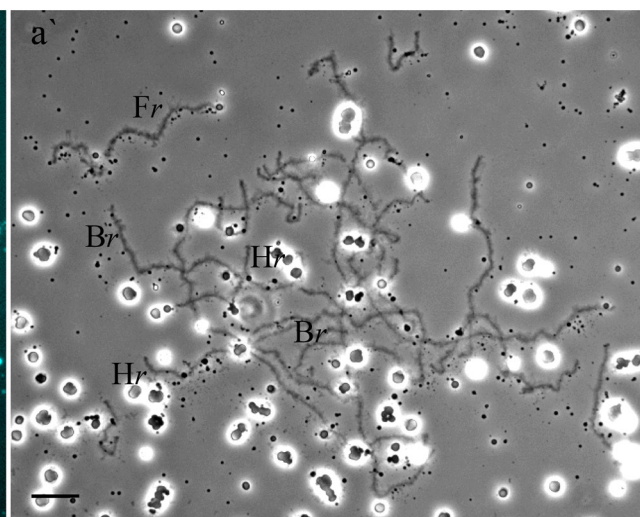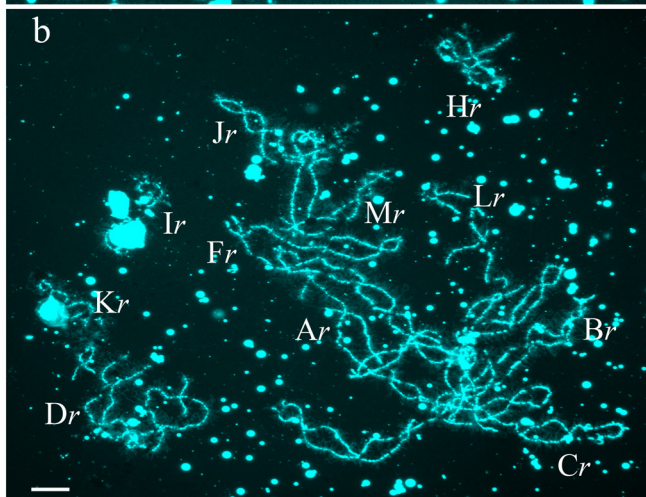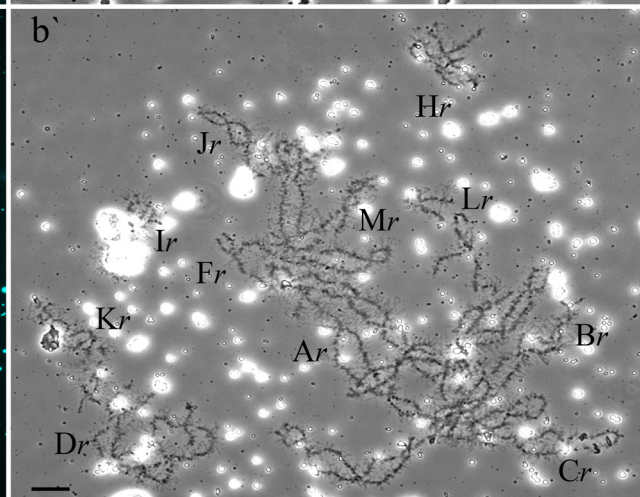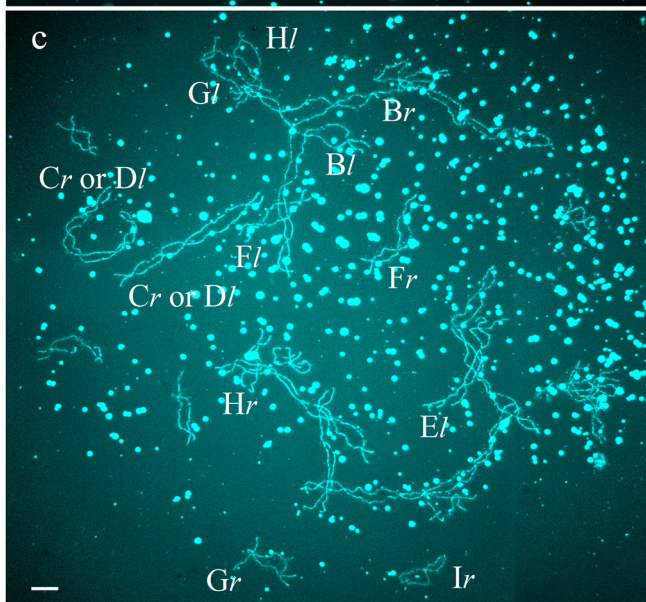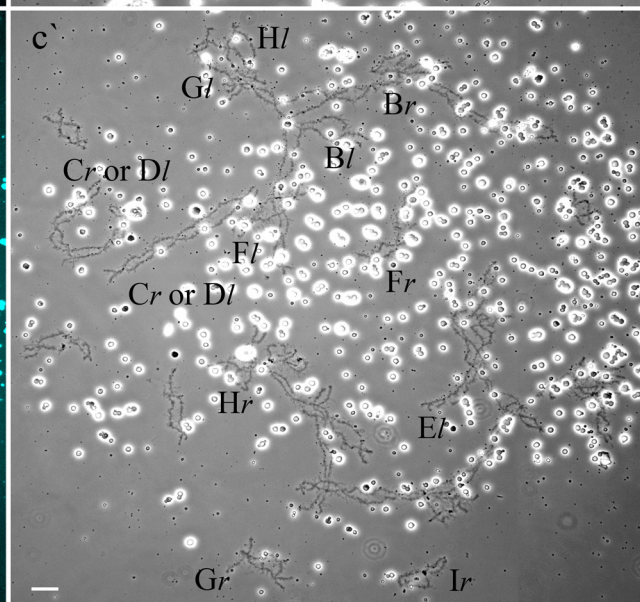

Supplement: S6 Fig — (a,a`) Lampbrush chromosome set from oocyte of diploid hybrid female represented by 26 univalents corresponding to P. ridibundus chromosomes. (b,b`) Lampbrush chromosome set from oocyte of diploid hybrid female represented by 13 bivalents corresponding to P. ridibundus chromosomes. (c,c`) Lampbrush chromosome set from oocyte of triploid hybrid female with LLR genotype represented by 26 bivalents, where 13 bivalents correspond to P. ridibundus chromosomes and 13 bivalents correspond to P. lessonae chromosomes. Letter symbols indicate alphabetic numbering of all lampbrush chromosomes; italic type shows correspondence of identified chromosomes to genotype of parental species: r—to P. ridibundus, l—to P. lessonae. Chromosomes were counterstained with DAPI. Corresponding phase-contrast micrographs are shown (a`,b`,c`). Scale bars = 50 μm. (PDF) [file pone.0123304.s006.pdf]

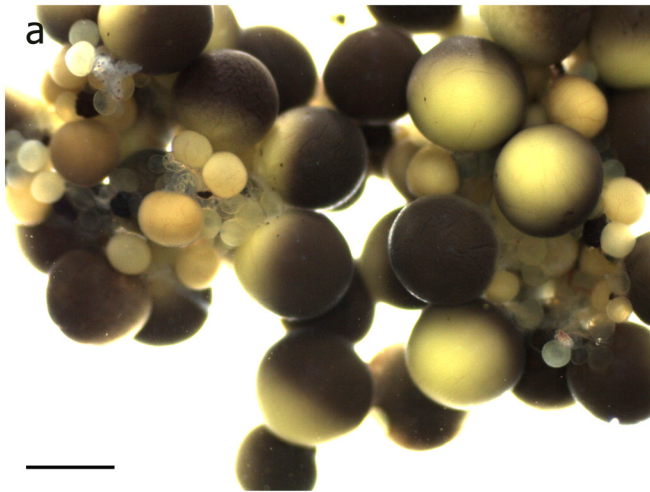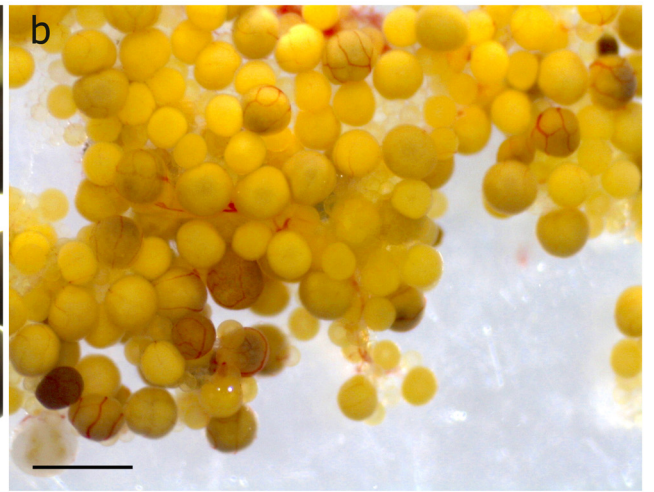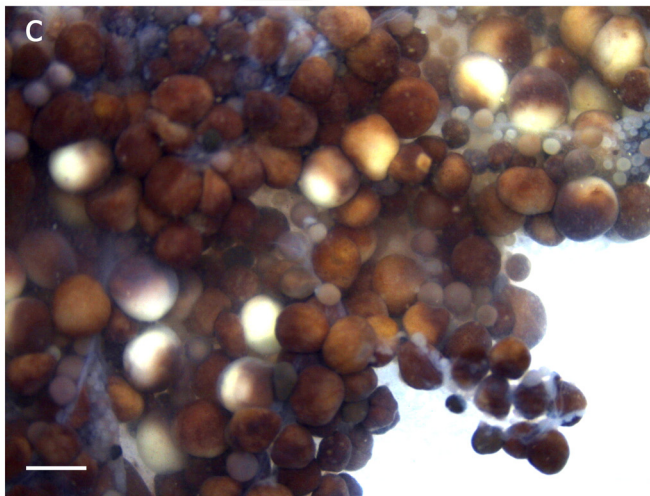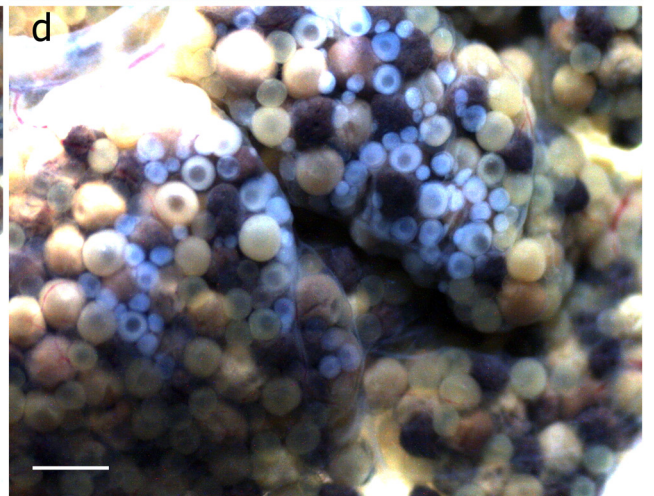

Supplement: S8 Fig — Ovary fragments of P. ridibundus (a), P. lessonae (b), triploid hybrid frog with LLR genotype (c) and diploid hybrid frog (d). Pre-vitellogenic, vitellogenic and post-vitellogenic oocytes (according to Dumont (1972) [*]) are present in the mature ovaries (a,c) but only pre- and vitellogenic oocytes are present in the immature ovaries (b,d). Ovaries of both parental species (a,b) are characterized by alive oocytes with regular rounded shape and a few dead oocytes. Ovaries of hybrid animals (c,d) have many dead oocytes with irregular shape and abnormal dark brown coloring of oocyte poles. Scale bars = 1 mm. * Dumont JN (1972) Oogenesis in Xenopus laevis (Daudin). I. Stages of oocyte development in laboratory maintained animals. J Morphol 136: 153–180. (PDF) [file pone.0123304.s008.pdf]

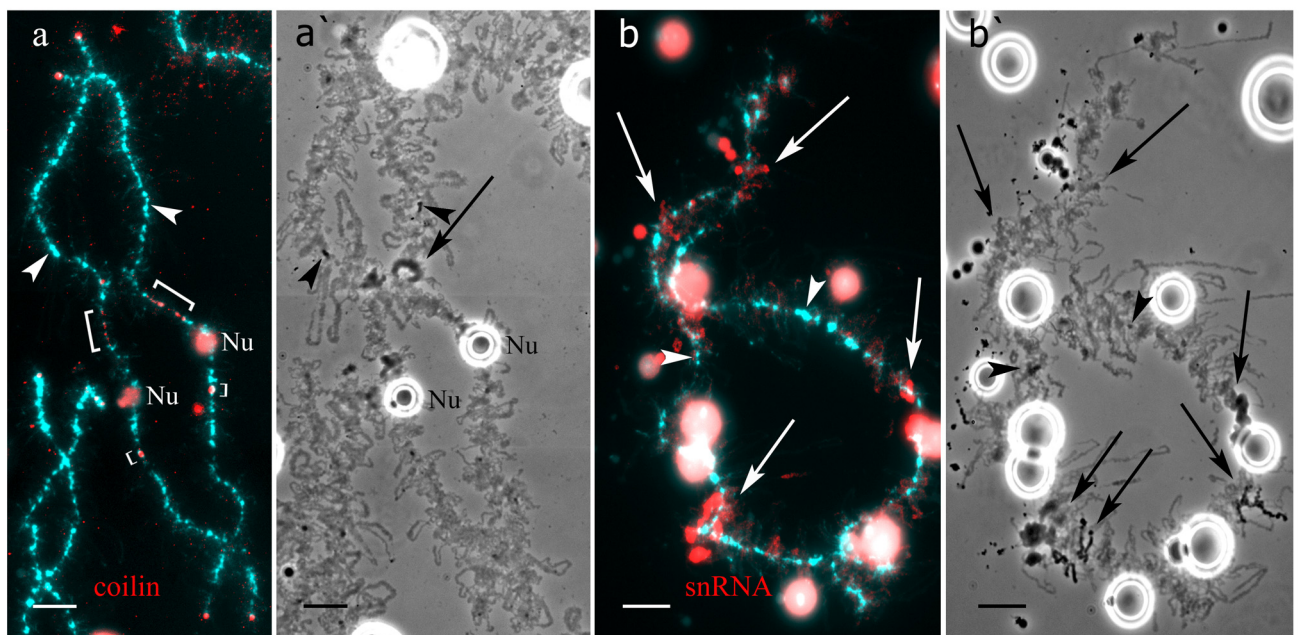

Supplement: S9 Fig — (a) Detection of chromosome-associated coilin-positive granules by immunofluorescent staining with R288 antibody. (b) Identification of marker loops enriched with splicing factors on lampbrush chromosome corresponding to chromosome I of P. ridibundus. Immunofluorescent staining with antibodies against TMG-cap of small nuclear RNA. Arrows show marker loops. Arrowheads indicate centromeres. Chromosomes were counterstained with DAPI. Corresponding phase-contrast micrographs are shown (a`,b`). Scale bars = 10 μm. (PDF) [file pone.0123304.s009.pdf]
