# Supplementary material for: Optional Endoreplication and Selective Elimination of Parental Genomes during Oogenesis in Diploid and Triploid Hybrid European Water Frogs
Source: PLoS One. 2015 Apr 20;10(4):e0123304. doi: 10.1371/journal.pone.0123304 (PMC4403867; doi:10.1371/journal.pone.0123304)
Supplement: S7 Fig — (a) During oogenesis of diploid hybrid frog only endoreplication of both parental genomes occurred to form oocytes with 26 bivalents (at the top), neither elimination nor endoreplication took place to form oocytes with 26 univalents (at the bottom). (b) During oogenesis of other diploid hybrid frog elimination of L genome (blue) and endoreplication of the remaining R genome (orange) occurred to form oocytes with 13 bivalents (at the top), elimination and endoreplication were omitted to form oocytes with 26 univalents (at the bottom). (c) During oogenesis of additional diploid hybrid frog neither elimination nor endoreplication occurred to form oocytes with 26 univalents (at the top). Losing of individual chromosomes corresponding to P. ridibundus chromosomes led to formation of aneuploid oocytes (in the middle). Endoreplication took place to form oocytes with 26 bivalents (at the bottom). (PDF) [file pone.0123304.s007.pdf]

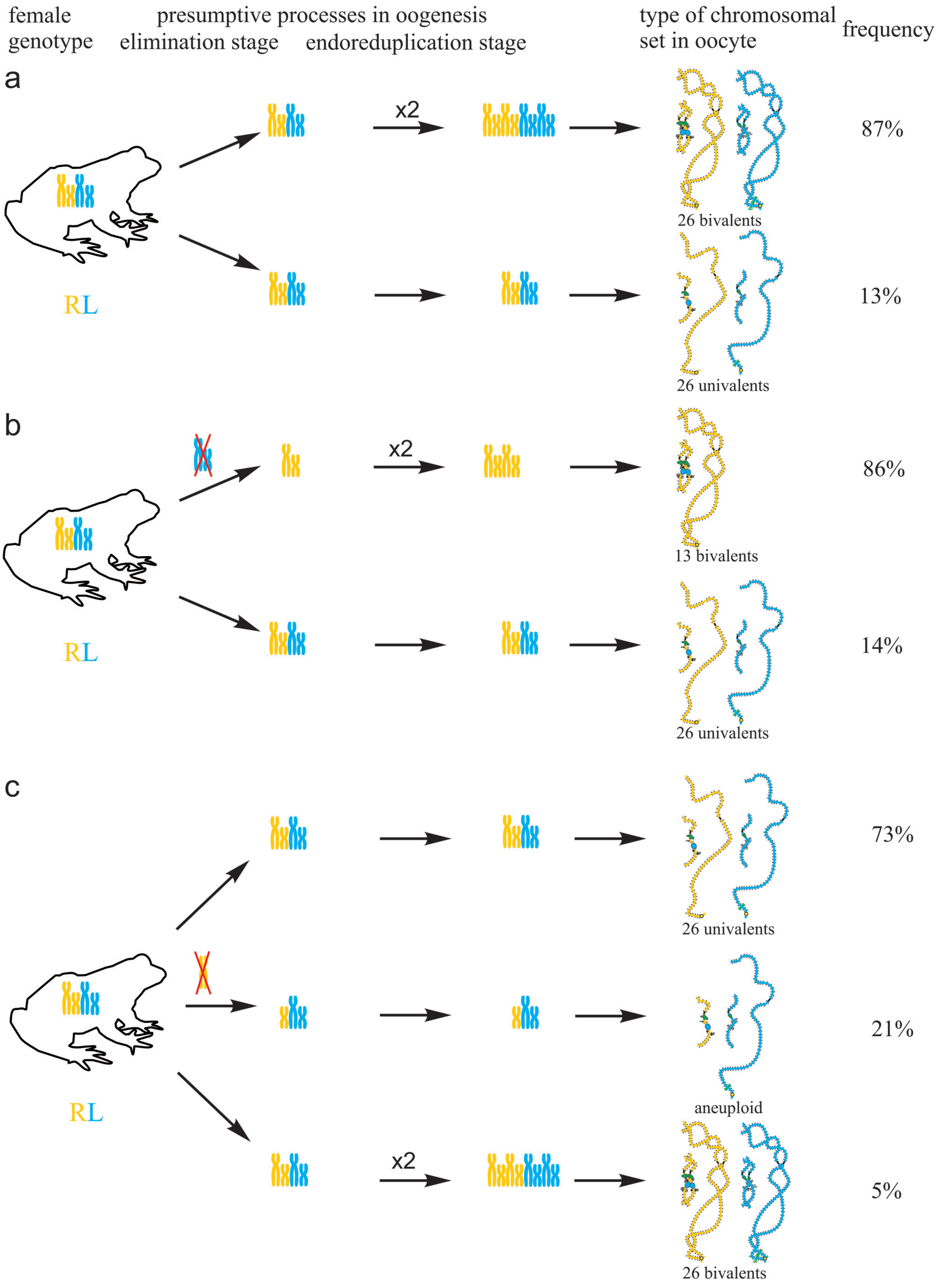
female genotype
presumptive processes in oogenesis
elimination stage
endoreduplication stage
type of chromosomal set in oocyte
frequency
a
x2
26 bivalents
87%
RL
26 univalents
13%
b
x2
13 bivalents
86%
RL
26 univalents
14%
c
26 univalents
73%
RL
aneuploid
21%
x2
26 bivalents
5%
