# Supplementary material for: Optional Endoreplication and Selective Elimination of Parental Genomes during Oogenesis in Diploid and Triploid Hybrid European Water Frogs
Source: PLoS One. 2015 Apr 20;10(4):e0123304. doi: 10.1371/journal.pone.0123304 (PMC4403867; doi:10.1371/journal.pone.0123304)
Supplement: S10 Fig — Triploid hybrids with RRL genotype produce oocytes with 13 bivalents corresponding to P. ridibundus chromosomes (at the top). Triploid females with LLR genotype produce oocytes with 13 bivalents corresponding to P. lessonae chromosomes, oocytes with 13 bivalents corresponding to P. ridibundus chromosomes and oocytes with 26 bivalents corresponding to both P. ridibundus and P. lessonae chromosomes (in the middle). Diploid hybrid frogs produce oocytes with 13 bivalents corresponding to P. ridibundus chromosomes, oocytes with 26 bivalents corresponding to both P. ridibundus and P. lessonae chromosomes and oocytes with 26 bivalents corresponding only to P. ridibundus chromosomes (at the bottom). (PDF) [file pone.0123304.s010.pdf]

Female genotype	Oocyte chromosomal set		
RRL	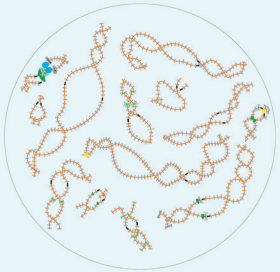 88,4%<br>13 bivalents of *P. ridibundus*		
LLR	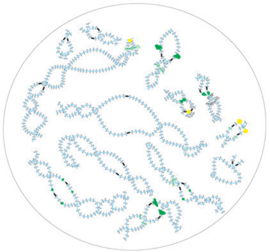 60%<br>13 bivalents of *P. lessonae*	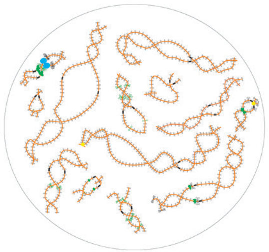 20%<br>13 bivalents of *P. ridibundus*	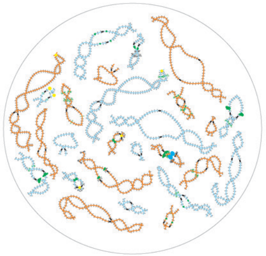 13,8%<br>26 bivalents:13 of *P. ridibundus,* 13 of *P. lessonae*
RL	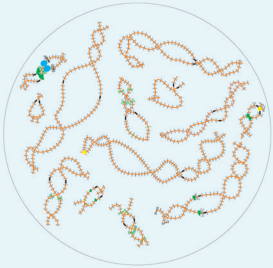 75,5%<br>13 bivalents of *P. ridibundus*	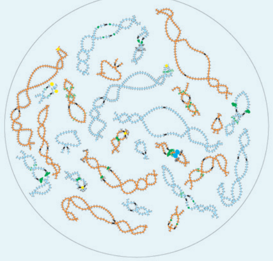 10,1%<br>26 bivalents:13 of *P. ridibundus,* 13 of *P. lessonae*	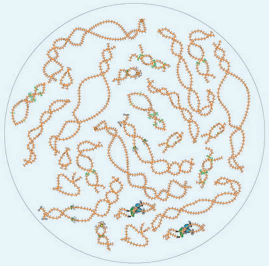 2,8%<br>26 bivalents of *P. ridibundus*
