# Supplementary material for: Optional Endoreplication and Selective Elimination of Parental Genomes during Oogenesis in Diploid and Triploid Hybrid European Water Frogs
Source: PLoS One. 2015 Apr 20;10(4):e0123304. doi: 10.1371/journal.pone.0123304 (PMC4403867; doi:10.1371/journal.pone.0123304)
Supplement: S1 Material — (DOC) [file pone.0123304.s011.doc]

**S1 Material. Supplementary Results. Description of ovaries from parental species and hybrid frogs and algorithm of lampbrush chromosome identification.**

**Morphology of ovaries from parental and hybrid species**

To evaluate fertility of hybrid and parental females we analysed ovaries according to Ogielska et al. [1]. In comparison to parental species ovaries of hybrid animals had many large perished oocytes which had irregular shape and lacked differential pole colouring. In some hybrid frogs, proportion of viable oocytes was about 20-30% from all mature oocytes; such oocytes can most likely become gametes (S8 Fig.).

**Chromosome sets from oocytes of hybrid frogs**

To study mechanisms of gametogenesis in di- and triploid hybrid females we analyzed chromosome sets from growing oocytes. The identification of lampbrush chromosomes (LBCs) obtained from oocytes of hybrids was based on their cytological maps available for both parental species individuals of close geographic origin [2]. In oocytes of hybrid frogs, marker structures on LBCs B, D, E, F, G, H and I were most reliable for morphological identification of *P. ridibundus* chromosomes (Figs. 1a,a` and 2d1-2d6`), while LBCs B, C, E, F, G, H and L were most reliable for morphological identification of *P. lessonae* chromosomes (Figs. 1c,c` and 2e1-2f1`). Other LBCs were unreliable for morphological identification of parental species karyotypes, since marker structures in these chromosomes either were formed in similar positions in both parental species or were highly polymorphic.

To simplify lampbrush chromosomes assignment to karyotype of one of the parental species, we identified landmark structures such as marker loops accumulating splicing factors and coilin-containing spheres and granules using immunofluorescent staining with antibodies against specific components (S9a,a`,b,b` Fig.). To confirm the cytological identification, FISH with probe to telomeric (TTAGGG)_n_ repeat was performed on lampbrush chromosome sets from oocytes of nearly all hybrid frogs. Mitotic chromosomes represented in Fig. 2a,b clearly demonstrate dissimilarities in interstitial telomere repeat sites (ITSs) on NOR-bearing chromosomes in parental species. Such differences enable to discriminate corresponding chromosomes in genomes of hybrid animals (Fig. 2c). In lampbrush chromosomes set analysis this approach allowed to identify and to assign accurately the NOR-bearing LBC H, which differs in two parental species in the positions of ITSs [2] (Fig. 2d6,e1).

Previously we found that chromosome associated nucleolus forms at the nucleolus organizer region on LBC H in oocytes of *P. ridibundus*but not *P. lessonae* from the Seversky Donets river basin, representing a promising marker for identification of the parental species karyotypes in hybrid frogs [2]. Here we demonstrate that in some hybrids, in oocytes where genomes of both parental species were present (oocytes with 26 and 39 uni- or bivalents), nucleolus appeared on lampbrush chromosome corresponding to *P. lessonae* LBC H (S1a6,a6`,b3,b3`,d2,d2`, S2a2 and S4a2,a2`,c2,c2` Figs. ). At the same time, nucleolus that formed on lampbrush chromosome corresponding to *P. ridibundus* LBC H was sometimes small and even undetectable (Fig. 1a4,a4`,b1,b1`; S4b3,b3`,c1,c1` Fig.). Inactivation of the nucleolus organizer regionin such oocytes seems to be accidental, but may be mediated by long or short noncoding RNAs transcribed from *P. lessonae* genome.

**Supplementary references**

1. Ogielska M, Rozenblut B, Augustynska R, Kotusz A (2010) Degeneration of germ line cells in amphibian ovary. Acta Zool Stockholm 91: 319–327.
2. Dedukh D, Mazepa G, Shabanov D, Rosanov J, Litvinchuk S, Borkin LJ, et al. (2013) Cytological maps of lampbrush chromosomes of European water frogs (*Pelophylax esculentus* complex) from the Eastern Ukraine. BMC Genet 14: 1–26.
