# Supplementary material for: Optional Endoreplication and Selective Elimination of Parental Genomes during Oogenesis in Diploid and Triploid Hybrid European Water Frogs
Source: PLoS One. 2015 Apr 20;10(4):e0123304. doi: 10.1371/journal.pone.0123304 (PMC4403867; doi:10.1371/journal.pone.0123304)
Supplement: S2 Material — (DOC) [file pone.0123304.s012.doc]

**Material S2. Supplementary Discussion. Mechanisms of hybrid frogs reproduction in the studied population systems of R-E type.**

We further evaluated the contribution of female gametes to the maintenance of the examined European water frog population systems of R-E type from Eastern Ukraine and suggested mechanisms of hybrid frogs reproduction (S10 Fig.). In S10 Fig. we represent all identified genomes transmitted in oocyte of diploid and triploid hybrid frogs which are supposed to form fertile gametes.

In *P. esculentus* population system of R-E type, oocytes with 13 bivalents produced by the majority of diploid, triploid RRL and one exceptional LLR hybrids can undergo meiosis and form haploid gametes containing *P. ridibundus* genome (S10 Fig.).Our results are opposite to previous studies of R-E population system from Poland and Germany where hybrid frogs which usually produce L gametes lead to hybrid frogs appearance after crossing with parental species (*P. ridibundus*) [1]–[3]. The majority of studied females with LLR genotypes could produce haploid gametes with *P. lessonae* genome. Such oocytes constitute about 60% among all analyzed oocytes from triploid frogs with LLR genotype (S10 Fig.). In addition, triploid frogs with LLR genotype produced oocytes with 13 bivalents corresponding to *P. ridibundus* chromosomes (20%) and oocytes with 26 bivalents corresponding to both *P. ridibundus* and *P. lessonae* genomes (about 14%).

According to our data the appearance of numerous diploid hybrid frogs in population system of R-E type is possible when haploid eggs with R genome are fertilized by the haploid sperm that contains L genome and was presumably produced by hybrid males, because mature *P. lessonae* individuals were not found in the examined population systems [4]. Fertilization of haploid egg with R genome by haploid sperm with R genome presumably gives rise to *P. ridibundus* parental species. Thus, in the examined population system of R-E type, genome composition presumably differs between haploid gametes in male and female hybrids.

We suppose that oocytes with 39 bivalents produced by triploid frogs are inessential for the maintenance of *P. esculentus* population systems in Eastern Ukraine since mature tetraploid hybrids were not reported in the population systems of R-E, L-E or E types. Furthermore, oocytes with univalents probably cannot overcome meiosis to produce fertile gametes. Oocytes with univalents and 39 bivalents composed about 12% among all observed oocytes produced by triploid frogs with RRL genotype (S10 Fig.). Several hybrid frogs produced oocytes with 26 bivalents where *P. ridibundus* and *P. lessonae* genomes or only *P. ridibundus* genome were present that constitute about 13% among all analyzed oocytes of diploid hybrid frogs (S10 Fig.). Such oocytes could complete meiosis and form diploid gametes with both *P. ridibundus* and *P. lessonae* genomes or two *P. ridibundus* genomes.

After fertilization by haploid sperm with R or L genomes [5], oocytes with 26 bivalents produced by some diploid *P. esculentus* may give rise to triploid RRL or LLR hybrids. Nevertheless, the number of diploid eggs with R or L genomes is presumably insufficient to sustain such a number of triploid hybrids in the population systems from Eastern Ukraine (up to 80% according to [6]). We conclude that diploid sperm is required to produce a large number of triploid hybrids in the analyzed populations of water frogs.

**Supplementary references**

1. Plötner, J (2005) Die westpaläarktichen Wasserfrösche. Bielefeld: Laurenti-Verlag. 1–161 p.
2. Graf JD, Polls-Pelaz M (1989) Evolutionary genetics of the *Rana esculenta* complex. In: Dawley RM, Bogart JP, editors. Evolution and ecology of unisexual vertebrates. Albany, New York: New York State Museum Publications. pp. 289–302.
3. Berger L (1990) On the origin of genetic systems in European water frog hybrids. Zool Pol 35(1–4): 5–32.
4. Korshunov AV (2009) Ecological patterns of biotopic distribution of *Pelophylax esculentus* complex in Kharkiv region. Kharkov Nat University J 8(828): 48–57.
5. Borkin LJ, Zinenko AI, Korshunov AV, Lada GA, Litvinchuk SN, Rosanov JM, et al. (2005) Mass polyploidy in *Rana esculenta* hybridogenic complex (Ranidae, Anura, Amphibia) in eastern Ukraine. Proceedings of the 1th Conference of the Ukrainian Herpetological Society. Kyiv. pp. 23–26.
6. Borkin LJ, Korshunov AV, Lada GA, Litvinchuk SN, Rosanov JM, Shabanov DA, et al. (2004) Mass occurrence of polyploid green frogs *(Rana esculenta* complex) in eastern Ukraine. Russ J Herpetol 11: 194–213.
