# Supplementary material for: Optional Endoreplication and Selective Elimination of Parental Genomes during Oogenesis in Diploid and Triploid Hybrid European Water Frogs
Source: PLoS One. 2015 Apr 20;10(4):e0123304. doi: 10.1371/journal.pone.0123304 (PMC4403867; doi:10.1371/journal.pone.0123304)
Supplement: S1 Table — C values—the amount of DNA per nucleus (genome size, in picograms, pg)—and genotypes are given for each female. (PDF) [file pone.0123304.s015.pdf]

Supplementary Table 1.
List of *P. esculentus* females from population systems of R-E type from the Seversky Donets river basin

Frog`s number	Mean of C	Female genotype	Locality	Number of analized oocytes	Oocyte chromosomal set	Comment
**1**	23.14	RRL	Dobritsky prud	1	13 bivalents P.ridibundus	
			Latitude 49°32'	2	13 bivalents P.ridibundus	
			Longitude 36°16'	3	13 bivalents P.ridibundus	
				4	13 bivalents P.ridibundus	
				5	13 bivalents P.ridibundus	
				6	13 bivalents P.ridibundus	
				7	13 bivalents P.ridibundus	
				8	13 bivalents P.ridibundus	
				9	13 bivalents P.ridibundus	
				10	13 bivalents P.ridibundus	
				11	13 bivalents P.ridibundus	
				12	13 bivalents P.ridibundus	
				13	13 bivalents P.ridibundus	
				14	13 bivalents P.ridibundus	
				15	13 bivalents P.ridibundus	
				16	13 bivalents P.ridibundus	
				17	13 bivalents P.ridibundus	
				18	13 bivalents P.ridibundus	
				19	13 bivalents P.ridibundus	
**2**	23.32	RRL	Dobritsky prud	1	13 bivalents P.ridibundus	
			Latitude 49°32'	2	13 bivalents P.ridibundus	
			Longitude 36°16'	3	13 bivalents P.ridibundus	
				4	13 bivalents P.ridibundus	
				5	13 bivalents P.ridibundus	

				6	13 bivalents P.ridibundus	
				7	13 bivalents P.ridibundus	
				8	13 bivalents P.ridibundus	
				9	13 bivalents P.ridibundus	
				10	13 bivalents P.ridibundus	
				11	13 bivalents P.ridibundus	
				12	13 bivalents P.ridibundus	
				13	13 bivalents P.ridibundus	
				14	13 bivalents P.ridibundus	
**3**	23.11	RRL	Dobritsky prud	1	13 bivalents P.ridibundus	
			Latitude 49°32'	2	13 bivalents P.ridibundus	
			Longitude 36°16'	3	13 bivalents P.ridibundus	
				4	13 bivalents P.ridibundus	
				5	13 bivalents P.ridibundus	
				6	13 bivalents P.ridibundus	
				7	13 bivalents P.ridibundus	
				8	13 bivalents P.ridibundus	
				9	13 bivalents P.ridibundus	
				10	13 bivalents P.ridibundus	
				11	13 bivalents P.ridibundus	
				12	13 bivalents P.ridibundus	
				13	13 bivalents P.ridibundus	
				14	13 bivalents P.ridibundus	
				15	13 bivalents P.ridibundus	
				16	13 bivalents P.ridibundus	
				17	13 bivalents P.ridibundus	
				18	13 bivalents P.ridibundus	
				19	13 bivalents P.ridibundus	
				20	13 bivalents P.ridibundus	
				21	13 bivalents P.ridibundus	
				22	13 bivalents P.ridibundus	

**4**	22.94	RRL	Dobritsky prud<br>Latitude 49°32'<br>Longitude 36°16'	1<br>2<br>3<br>4<br>5<br>6<br>7<br>8<br>9<br>10<br>11<br>12<br>13<br>14<br>15<br>16<br>17<br>18<br>19<br>20<br>21<br>22<br>23<br>24<br>25	13 bivalents P.ridibundus<br>13 bivalents P.ridibundus	
**5**	23.25	RRL	Dobritsky prud<br>Latitude 49°32'<br>Longitude 36°16'	1<br>2<br>3<br>4<br>5	13 bivalents P.ridibundus<br>13 bivalents P.ridibundus<br>13 bivalents P.ridibundus<br>13 bivalents P.ridibundus<br>13 bivalents P.ridibundus	

				6	13 bivalents P.ridibundus	
				7	13 bivalents P.ridibundus	
				8	13 bivalents P.ridibundus	
				9	13 bivalents P.ridibundus	
				10	13 bivalents P.ridibundus	
				11	13 bivalents P.ridibundus	
				12	13 bivalents P.ridibundus	
				13	13 bivalents P.ridibundus	
				14	13 bivalents P.ridibundus	
				15	13 bivalents P.ridibundus	
				16	13 bivalents P.ridibundus	
				17	13 bivalents P.ridibundus	
				18	13 bivalents P.ridibundus	
**6**	15,26	RL	Dobritsky prud	1	26 bivalents 13 P.ridibundus, 13 P.lessonae	
			Latitude 49°32'	2	26 bivalents 13 P.ridibundus, 13 P.lessonae	
			Longitude 36°16'	3	26 bivalents 13 P.ridibundus, 13 P.lessonae	
				4	26 bivalents 13 P.ridibundus, 13 P.lessonae	
				5	26 bivalents 13 P.ridibundus, 13 P.lessonae	
				6	26 bivalents 13 P.ridibundus, 13 P.lessonae	
				7	26 bivalents 13 P.ridibundus, 13 P.lessonae	
				8	26 bivalents 13 P.ridibundus, 13 P.lessonae	
				9	26 univalents 13 P.ridibundus, 13 P.lessonae	
				10	26 univalents 13 P.ridibundus, 13 P.lessonae	
				11	26 bivalents 13 P.ridibundus, 13 P.lessonae	
				12	26 bivalents 13 P.ridibundus, 13 P.lessonae	
				13	26 bivalents 13 P.ridibundus, 13 P.lessonae	
				14	26 bivalents 13 P.ridibundus, 13 P.lessonae	
				15	26 bivalents 13 P.ridibundus, 13 P.lessonae	FISH with TTAGGG
				16	26 bivalents 13 P.ridibundus, 13 P.lessonae	FISH with TTAGGG
				17	26 bivalents 13 P.ridibundus, 13 P.lessonae	FISH with TTAGGG
				18	26 bivalents 13 P.ridibundus, 13 P.lessonae	FISH with TTAGGG

				19	26 bivalents 13 P.ridibundus, 13 P.lessonae	
				20	26 bivalents 13 P.ridibundus, 13 P.lessonae	
				21	26 univalalents 13 P.ridibundus, 13 P.lessonae	
				22	26 bivalents 13 P.ridibundus, 13 P.lessonae	
				23	26 bivalents 13 P.ridibundus, 13 P.lessonae	
**7**	22,84	RRL	Pojma (П159)	1	13 bivalents P.ridibundus	
			Latitude 49°38'	2	13 bivalents P.ridibundus	
			Longitude 36°20'	3	13 bivalents P.ridibundus	
				4	13 bivalents P.ridibundus	
				5	13 bivalents P.ridibundus	
				6	13 bivalents P.ridibundus	
				7	13 bivalents P.ridibundus	
				8	13 bivalents P.ridibundus	
				9	13 bivalents P.ridibundus	
				10	13 bivalents P.ridibundus	
				11	13 bivalents P.ridibundus	
				12	13 bivalents P.ridibundus	
				13	13 bivalents P.ridibundus	
				14	13 bivalents P.ridibundus	
				15	13 bivalents P.ridibundus	
				16	13 bivalents P.ridibundus	
				17	13 bivalents P.ridibundus	
				18	13 bivalents P.ridibundus	
				19	13 bivalents P.ridibundus	
				20	13 bivalents P.ridibundus	
**8**	23,01	RRL	Pojma (П188)	1	13 bivalents P.ridibundus	
			Latitude 49°38'	2	13 bivalents P.ridibundus	
			Longitude 36°20'	3	13 bivalents P.ridibundus	
				4	13 bivalents P.ridibundus	
				5	13 bivalents P.ridibundus	

				6	13 bivalents P.ridibundus	
				7	13 bivalents P.ridibundus	
				8	13 bivalents P.ridibundus	
				9	13 bivalents P.ridibundus	
				10	13 bivalents P.ridibundus	
				11	13 bivalents P.ridibundus	
				12	13 bivalents P.ridibundus	
				13	13 bivalents P.ridibundus	
				14	13 bivalents P.ridibundus	
				15	13 bivalents P.ridibundus	
				16	13 bivalents P.ridibundus	
				17	13 bivalents P.ridibundus	
				18	13 bivalents P.ridibundus	
				19	13 bivalents P.ridibundus	
				20	13 bivalents P.ridibundus	
**9**	22,15	LRx	Pojma (П162)	1	39 univalents	
			Latitude 49°38'	2	39 univalents	
			Longitude 36°20'	3	39 bivalents 26 P.ridibundus, 13 P.lessonae	FISH with TTAGGG
				4	39 univalents 26 P.ridibundus, 13 P.lessonae	
				5	39 univalents	
				6	39 univalents 26 P.ridibundus, 13 P.lessonae	
				7	39 univalents	
				8	39 univalents	
				9	39 univalents	
				10	39 univalents	
				11	39 univalents	
				12	39 univalents 26 P.ridibundus, 13 P.lessonae	
				13	39 univalents	
				14	39 univalents	
				15	39 univalents	
				16	39 univalents	

				17	39 univalents	
				18	39 univalents	
				19	39 univalents	
				20	aneuploid, bi- and univalents	FISH with TTAGGG
				21	39 univalents	
				22	39 univalents	
				23	39 univalents	
				24	39 univalents	
				25	39 univalents	
				26	39 univalents	
				27	39 univalalents 26 P.ridibundus, 13 P.lessonae	
				28	39 univalents	
				29	39 univalents	
				30	39 univalalents 26 P.ridibundus, 13 P.lessonae	
				31	39 univalalents 26 P.ridibundus, 13 P.lessonae	FISH with TTAGGG
				32	39 univalalents 26 P.ridibundus, 13 P.lessonae	FISH with TTAGGG
				33	39 univalalents 26 P.ridibundus, 13 P.lessonae	FISH with TTAGGG
				34	39 univalents	
				35	39 univalents	
				36	39 bivalents 26 P.ridibundus, 13 P.lessonae	FISH with TTAGGG
				37	39 bivalents 26 P.ridibundus, 13 P.lessonae	FISH with TTAGGG
				38	39 bivalents 26 P.ridibundus, 13 P.lessonae	FISH with TTAGGG
				39	39 univalents	FISH with TTAGGG
**10**	22,35	LRx	Pojma (П165)<br>Latitude 49°38'<br>Longitude 36°20'	1	13 bivalents P.ridibundus, 13 univalents P.lessona	FISH with TTAGGG
				2	13 bivalents P.ridibundus	
				3	13 bivalents P.ridibundus	
				4	39 bivalents 26 P.ridibundus, 13 P.lessonae	FISH with TTAGGG
				5	13 bivalents P.ridibundus	FISH with TTAGGG
				6	13 bivalents P.ridibundus, 13 univalents P.lessona	FISH with TTAGGG
				7	13 bivalents P.ridibundus, 13 univalents P.lessona	FISH with TTAGGG
				8	13 bivalents P.ridibundus, 13 univalents P.lessona	FISH with TTAGGG

				9	13 bivalents P.ridibundus, 13 univalents P.lessona	FISH with TTAGGG
				10	13 bivalents P.ridibundus, 13 univalents P.lessona	FISH with TTAGGG
				11	13 bivalents P.ridibundus	FISH with TTAGGG
				12	13 bivalents P.ridibundus	
				13	13 bivalents P.ridibundus, 13 univalents P.lessonae	
				14	13 bivalents P.ridibundus, 13 univalents P.lessonae	
				15	13 bivalents P.ridibundus	
				16	13 bivalents P.ridibundus, 13 univalents P.lessonae	
				17	13 bivalents P.ridibundus	
				18	13 bivalents P.ridibundus, 13 univalents P.lessonae	
				19	13 bivalents P.ridibundus, 13 univalents P.lessonae	
				20	13 bivalents P.ridibundus, 13 univalents P.lessonae	
				21	13 bivalents P.ridibundus, 13 univalents P.lessonae	
				22	13 bivalents P.ridibundus, 13 univalents P.lessonae	
				23	13 bivalents P.ridibundus	
				24	13 bivalents P.ridibundus	
				25	13 bivalents P.ridibundus	
				26	13 bivalents P.ridibundus	
				27	13 bivalents P.ridibundus, 13 univalents P.lessonae	
				28	13 bivalents P.ridibundus	
				29	13 bivalents P.ridibundus	
**11**	15,17	RL	Pojma (П168)	1	26 bivalents P.ridibundus	FISH with TTAGGG
			Latitude 49°38'	2	26 univalents P.ridibundus	FISH with TTAGGG
			Longitude 36°20'	3	26 bivalents P.ridibundus	FISH with TTAGGG
				4	26 univalents P.ridibundus	FISH with TTAGGG
				5	26 univalents P.ridibundus	FISH with TTAGGG
				6	26 bivalents P.ridibundus	FISH with TTAGGG
				7	26 bivalents P.ridibundus	FISH with TTAGGG
				8	26 univalents P.ridibundus	FISH with TTAGGG
				9	26 bivalents P.ridibundus	FISH with TTAGGG
				10	4 bivalents, 15 univalents P.ridibundus	FISH with TTAGGG

				11	6 bivalents, 14 univalents P.ridibundus	FISH with TTAGGG
				12	3 bivalents, 19 univalents P.ridibundus	FISH with TTAGGG
				13	3 bivalents, 16 univalents P.ridibundus	
				14	4 bivalents, 15 univalents P.ridibundus	
				15	10 bivalents, 6 unival, P.ridibundus	
				16	26 bivalents P.ridibundus	
				17	26 bivalents P.ridibundus	
				18	4 bivalents, 14 univalents P.ridibundus	
				19	3 bivalents, 15 univalents P.ridibundus	
				20	5 bivalents, 10 univalents P.ridibundus	
				21	3 bivalents, 19 univalents P.ridibundus	
				22	5 bivalents, 16 univalents P.ridibundus	
				23	5 bivalents, 16 univalents P.ridibundus	
				24	5 bivalents, 15 univalents P.ridibundus	
				25	3 bivalents, 29 univalents P.ridibundus	
				26	26 bivalents P.ridibundus	
				27	26 bivalents P.ridibundus	
				28	26 univalents P.ridibundus	
				29	4 bivalents, 18univalents P.ridibundus	
				30	26 univalents P.ridibundus	
				31	3 bivalents, 19 univalents P.ridibundus	
**12**	20,28	RLL	Iskov prud	1	26 univalents 13 P.ridibundus, 13 P.lessonae	
			Latitude 49°33'	2	26 univalents 13 P.ridibundus, 13 P.lessonae	
			Longitude 36°17'	3	26 bivalents 13 P.ridibundus, 13 P.lessonae	
				4	26 univalents 13 P.ridibundus, 13 P.lessonae	FISH with TTAGGG
				5	26 bivalents 13 P.ridibundus, 13 P.lessonae	FISH with TTAGGG
				6	26 bivalents 13 P.ridibundus, 13 P.lessonae	FISH with TTAGGG
				7	26 bivalents 13 P.ridibundus, 13 P.lessonae	
				8	26 bivalents 13 P.ridibundus, 13 P.lessonae	
				9	26 univalents 13 P.ridibundus, 13 P.lessonae	
				10	26 bivalents 13 P.ridibundus, 13 P.lessonae	

				11	26 bivalents 13 P.ridibundus, 13 P.lessonae	
				12	26 bivalents 13 P.ridibundus, 13 P.lessonae	
				13	26 bivalents 13 P.ridibundus, 13 P.lessonae	
				14	26 bivalents 13 P.ridibundus, 13 P.lessonae	
				15	26 univalents 13 P.ridibundus, 13 P.lessonae	
				16	26 bivalents 13 P.ridibundus, 13 P.lessonae	
**13**	14,65	RL	Iskov prud	1	13 bivalents P.ridibundus	
			Latitude 49°33'	2	13 bivalents P.ridibundus	
			Longitude 36°17'	3	13 bivalents P.ridibundus	
				4	13 bivalents P.ridibundus	
				5	26 univalents 13 P.ridibundus, 13 P.lessonae	
				6	13 bivalents P.ridibundus	
				7	13 bivalents P.ridibundus	
**14**	14,92	RL	Iskov prud (И5)	1	13 bivalents P.ridibundus	
			Latitude 49°33'	2	13 bivalents P.ridibundus	
			Longitude 36°17'	3	13 bivalents P.ridibundus	
				4	13 bivalents P.ridibundus	
				5	13 bivalents P.ridibundus	
				6	13 bivalents P.ridibundus	
				7	13 bivalents P.ridibundus	
				8	13 bivalents P.ridibundus	
				9	13 bivalents P.ridibundus	
				10	13 bivalents P.ridibundus	
				11	13 bivalents P.ridibundus	
				12	13 bivalents P.ridibundus	
				13	13 bivalents P.ridibundus	
				14	13 bivalents P.ridibundus	
				15	13 bivalents P.ridibundus	
				16	13 bivalents P.ridibundus	
				17	13 bivalents P.ridibundus	

				18	13 bivalents P.ridibundus	immunostaining against coilin
				19	13 bivalents P.ridibundus	immunostaining against coilin
				20	13 bivalents P.ridibundus	immunostaining against coilin
				21	13 bivalents P.ridibundus	
				22	13 bivalents P.ridibundus	immunostaining with K 121
				23	13 bivalents P.ridibundus	immunostaining with K 121
**15**	22,88	RRL	Sykhaja Gomolsha (F	1	13 bivalents P.ridibundus	
			Latitude 49°32'	2	13 bivalents P.ridibundus	
			Longitude 36°20'	3	13 bivalents P.ridibundus	
				4	13 bivalents P.ridibundus	
				5	13 bivalents P.ridibundus	
**16**	22,68	RRL	Sykhaja Gomolsha (4	1	13 bivalents P.ridibundus	
			Latitude 49°32'	2	13 bivalents P.ridibundus	
			Longitude 36°20'	3	13 bivalents P.ridibundus	
				4	13 bivalents P.ridibundus	
				5	13 bivalents P.ridibundus	
				6	13 bivalents P.ridibundus	
				7	13 bivalents P.ridibundus	
				8	13 bivalents P.ridibundus	
				9	13 bivalents P.ridibundus	
				10	13 bivalents P.ridibundus	
				11	13 bivalents P.ridibundus	
				12	13 bivalents P.ridibundus	
				13	13 bivalents P.ridibundus	
				14	13 bivalents P.ridibundus	
				15	13 bivalents P.ridibundus	
				16	13 bivalents P.ridibundus	
**17**	22,75	RRL	Sykhaja Gomolsha (F	1	13 bivalents P.ridibundus	
			Latitude 49°32'	2	13 bivalents P.ridibundus	

			Longitude 36°20'	3	13 bivalents P.ridibundus	
				4	13 bivalents P.ridibundus	
				5	13 bivalents P.ridibundus	
				6	13 bivalents P.ridibundus	
				7	13 bivalents P.ridibundus	
				8	13 bivalents P.ridibundus	
				9	13 bivalents P.ridibundus	
				10	13 bivalents P.ridibundus	
				11	13 bivalents P.ridibundus	
				12	13 bivalents P.ridibundus	
				13	13 bivalents P.ridibundus	
				14	13 bivalents P.ridibundus	
				15	13 bivalents P.ridibundus	
				16	13 bivalents P.ridibundus	
				17	13 bivalents P.ridibundus	
				18	13 bivalents P.ridibundus	
				19	13 bivalents P.ridibundus	
				20	13 bivalents P.ridibundus	
				21	13 bivalents P.ridibundus	
				22	13 bivalents P.ridibundus	
				23	13 bivalents P.ridibundus	
				24	13 bivalents P.ridibundus	
**18**	23,01	RRL	Sykhaja Gomolsha ((	1	13 bivalents P.ridibundus	
			Latitude 49°32'	2	13 bivalents P.ridibundus	
			Longitude 36°20'	3	13 bivalents P.ridibundus	
				4	13 bivalents P.ridibundus	
				5	13 bivalents P.ridibundus	
				6	13 bivalents P.ridibundus	
				7	13 bivalents P.ridibundus	
				8	13 bivalents P.ridibundus	
				9	13 bivalents P.ridibundus	

				10	13 bivalents P.ridibundus	
**19**	15,01	RL	Sykhaja Gomolsha (C<br>Latitude 49°32'<br>Longitude 36°20'	1	13 bivalents P.ridibundus	
				2	13 bivalents P.ridibundus	
				3	13 bivalents P.ridibundus	
				4	13 bivalents P.ridibundus	
**20**	14,91	RL	Sykhaja Gomolsha (2<br>Latitude 49°32'<br>Longitude 36°20'	1	13 bivalents P.ridibundus	
				2	13 bivalents P.ridibundus	immunostaining against coilin
				3	13 bivalents P.ridibundus	immunostaining against coilin
				4	13 bivalents P.ridibundus	immunostaining against coilin
				5	13 bivalents P.ridibundus	
**21**	15,02	RL	Sykhaja Gomolsha (F<br>Latitude 49°32'<br>Longitude 36°20'	1	13 bivalents P.ridibundus	
				2	13 bivalents P.ridibundus	
				3	13 bivalents P.ridibundus	
				4	13 bivalents P.ridibundus	
				5	13 bivalents P.ridibundus	
				6	13 bivalents P.ridibundus	
				7	13 bivalents P.ridibundus	
				8	13 bivalents P.ridibundus	
				9	13 bivalents P.ridibundus	
				10	13 bivalents P.ridibundus	
				11	13 bivalents P.ridibundus	
				12	13 bivalents P.ridibundus	
				13	13 bivalents P.ridibundus	
				14	13 bivalents P.ridibundus	
				15	13 bivalents P.ridibundus	
				16	13 bivalents P.ridibundus	
				17	13 bivalents P.ridibundus	
				18	13 bivalents P.ridibundus	

**22**	21,8	RLL	Zhovtnevoe (f4)<br>Latitude 50°11'<br>Longitude 36° 24′	1<br>2<br>3<br>4<br>5<br>6<br>7<br>8<br>9<br>10<br>11	13 bivalents P.lessonae<br>13 bivalents P.lessonae<br>13 bivalents P.lessonae<br>13 bivalents P.lessonae<br>13 bivalents P.lessonae<br>13 bivalents P.lessonae<br>13 bivalents P.lessonae<br>13 bivalents P.lessonae<br>13 bivalents P.lessonae<br>13 bivalents P.lessonae<br>13 bivalents P.lessonae	
**23**	14,76	RL	Zhovtnevoe (f1)<br>Latitude 50°11'<br>Longitude 36° 24′	2<br>3<br>4<br>5<br>6<br>7<br>8<br>9<br>10<br>11<br>12<br>13	13 bivalents<br>13 bivalents<br>13 bivalents P.ridibundus<br>13 bivalents<br>13 bivalents P.ridibundus<br>13 bivalents P.ridibundus<br>13 bivalents P.ridibundus<br>13 bivalents P.ridibundus<br>13 bivalents P.ridibundus<br>13 bivalents P.ridibundus<br>13 bivalents P.ridibundus<br>13 bivalents P.ridibundus	
**24**	22,04	RLL	Zhovtnevoe (g4)<br>Latitude 50°11'<br>Longitude 36° 24′	1<br>2<br>3<br>4<br>5<br>6<br>7	13 bivalents P.ridibundus<br>13 bivalents P.ridibundus<br>13 bivalents P.ridibundus<br>13 bivalents P.ridibundus<br>13 bivalents P.ridibundus<br>13 bivalents P.ridibundus<br>13 bivalents P.ridibundus	

				8	13 bivalents P.ridibundus	
				9	13 bivalents P.ridibundus	
				10	13 bivalents P.ridibundus	
				11	13 bivalents P.ridibundus	
				12	13 bivalents P.ridibundus	
				13	13 bivalents P.ridibundus	
				14	13 bivalents P.ridibundus	
				15	13 bivalents P.ridibundus	
				16	13 bivalents P.ridibundus	
				17	13 bivalents P.ridibundus	
				18	13 bivalents P.ridibundus	
				19	13 bivalents P.ridibundus	
				20	13 bivalents P.ridibundus	
				21	13 bivalents P.ridibundus	
				22	13 bivalents P.ridibundus	
				23	13 bivalents P.ridibundus	
**25**	21,87	RLL	Krasnoyarskoe Lake	1	13 bivalents P.lessonae	
			Latitude 49°01'	2	13 bivalents P.lessonae	
			Longitude 37°68'	3	13 bivalents P.lessonae	
				4	13 bivalents P.lessonae	
				5	13 bivalents P.lessonae	
				6	13 bivalents P.lessonae	
				7	13 bivalents P.lessonae	
				8	13 bivalents P.lessonae	
				9	13 bivalents P.lessonae	
**26**	21,73	RLL	Krasnoyarskoe Lake	1	13 bivalents	
			Latitude 49°01'	2	13 bivalents	
			Longitude 37°68'	3	13 bivalents	
				4	13 bivalents	
				5	13 bivalents P.lessonae	

				6	13 bivalents P.lessonae	
				7	13 bivalents P.lessonae	
				8	13 bivalents P.lessonae	
				9	13 bivalents P.lessonae	
				10	13 bivalents P.lessonae	
				11	13 bivalents P.lessonae	
**27**	15,06	RL	Krasnoyarskoe Lake	1	26 univalents 13 P.ridibundus, 13 P.lessonae	
			Latitude 49°01'	2	26 univalents 13 P.ridibundus, 13 P.lessonae	
			Longitude 37°68'	3	26 univalents 13 P.ridibundus, 13 P.lessonae	
				4	26 univalents 13 P.ridibundus, 13 P.lessonae	
				5	26 univalents 13 P.ridibundus, 13 P.lessonae	
				6	26 univalents 13 P.ridibundus, 13 P.lessonae	
				7	aneuploid, 20 univalents	
				8	26 univalents 13 P.ridibundus, 13 P.lessonae	
				9	26 univalents 13 P.ridibundus, 13 P.lessonae	
				10	26 univalents 13 P.ridibundus, 13 P.lessonae	
				11	26 univalents 13 P.ridibundus, 13 P.lessonae	
				12	aneuploid, 15 univalents	
				13	26 univalents 13 P.ridibundus, 13 P.lessonae	
				14	aneuploid, 20 univalents	
				15	26 univalents 13 P.ridibundus, 13 P.lessonae	
				16	26 univalents 13 P.ridibundus, 13 P.lessonae	
				17	26 univalents 13 P.ridibundus, 13 P.lessonae	
				18	26 univalents 13 P.ridibundus, 13 P.lessonae	FISH with TTAGGG
				19	26 univalents 13 P.ridibundus, 13 P.lessonae	FISH with TTAGGG
				20	26 univalents 13 P.ridibundus, 13 P.lessonae	FISH with TTAGGG
				21	26 univalents 13 P.ridibundus, 13 P.lessonae	immunostaining with K 121
				22	aneuploid, 18 univalents	immunostaining with K 121
				23	26 bivalents 13 P.ridibundus, 13 P.lessonae	immunostaining with K 121
				24	26 univalents 13 P.ridibundus, 13 P.lessonae	immunostaining with K 121
				25	aneuploid, 18 univalents	

				26	26 univalents 13 P.ridibundus, 13 P.lessonae	immunostaining against coilin
				27	26 univalents 13 P.ridibundus, 13 P.lessonae	
				28	26 univalents 13 P.ridibundus, 13 P.lessonae	
				29	26 univalents 13 P.ridibundus, 13 P.lessonae	
				30	26 univalents 13 P.ridibundus, 13 P.lessonae	
				31	26 univalents 13 P.ridibundus, 13 P.lessonae	
				32	aneuploid, 23 univalents	
				33	26 univalents 13 P.ridibundus, 13 P.lessonae	
				34	26 univalents 13 P.ridibundus, 13 P.lessonae	
				35	aneuploid, 20 univalents	
				36	26 bivalents 13 P.ridibundus, 13 P.lessonae	
				37	aneuploid, 20 univalents	
				38	26 univalents 13 P.ridibundus, 13 P.lessonae	
				39	26 univalents 13 P.ridibundus, 13 P.lessonae	
				40	aneuploid, 22 univalents	

Legend

(green)	very good chromosomal preparations. All chromosomes lay separately and they are clearly identified
(white)	good chromosomal prepararions. Individual chromosomes can be overlaped or stretched, nearly all chromosomes are well identified
(yellow)	chromosomal preparations of average quality. Some chromosomes are stretched or destroyed, the majority chromosomes are identified
